# Inhibition of transition metals dissolution in cobalt-free cathode with ultrathin robust interphase in concentrated electrolyte

**DOI:** 10.1038/s41467-020-17396-x

**Published:** 2020-07-20

**Authors:** Wei Liu, Jinxing Li, Wenting Li, Hanying Xu, Chao Zhang, Xinping Qiu

**Affiliations:** 1 Key Laboratory of Organic Optoelectronics and Molecular Engineering, Department of Chemistry, Tsinghua University, 100084 Beijing, China; 2 School of Metallurgical and Ecological Engineering, University of Science and Technology Beijing, 100083 Beijing, China

**Keywords:** Batteries, Batteries, Batteries

## Abstract

The low Coulombic efficiency during cycling hinders the application of Cobalt-free lithium-rich materials in lithium-ion batteries. Here we demonstrated that the dissolution of iron, rather than traditionally acknowledged manganese, is mainly responsible for the low Coulombic efficiency of the iron-substituted cobalt-free lithium-rich material. Besides, we presented an approach to inhibit the dissolution of transition metal ions by using concentrated electrolytes. We found that the cathode electrolyte interphase (CEI) layer formed in the concentrated electrolyte is a uniform and robust LiF-rich CEI, which is a sharp contrast with the uneven and fragile organic-rich CEI formed in the dilute electrolyte. The LiF-rich CEI not only effectively inhibits the dissolution of TMs but also stabilizes the cathode structure. The Coulombic efficiency, cycling stability, rate performance, and safety of the Fe-substituted cobalt-free lithium-rich cathode material in the concentrated electrolyte have been improved tremendously.

## Abstract (web summary)

The transition metal dissolution of cathode materials during cycling limits battery performances and lifetimes. Here, authors inhibit metal dissolution by using highly concentrated electrolytes that induce ultra-thin cathode electrolyte interphase formation on cathode surfaces during cycling.

## Introduction

Rechargeable lithium-ion batteries (LIBs) are becoming promising energy storage devices for electric vehicles (EVs) due to their high energy density and long life characteristics^1–4^. However, traditional cathode materials, such as layered LiCoO_2_, LiNi_*x*_Co_*y*_Mn_1-*x*-*y*_O_2_, and Olivine-type LiFePO_4_, exhibit insufficient discharge-specific capacities (<180 mAh g^−1^) and do not meet the requirements for EVs^5–8^. Hence, the development of new cathode materials with higher energy densities is crucial. Lithium-rich materials, normally denoted as *x*Li_2_MnO_3_·(1 − *x*)LiMO_2_ (M = Ni, Co, Mn, Fe, Al, Cr, etc.), can deliver high discharge-specific energy (250 mAh g^−1^) and have attracted much attention in recent years^5,9–11^. Nevertheless, some drawbacks, such as poor cycle performance, fast voltage fade, undesirable rate performance, and low coulombic efficiency (CE), still hinder the practical application of Li-rich materials^11,12^. It is widely believed that the continuous decomposition of the electrolyte and the deterioration of the structure of Li-rich materials during cycling are the main causes of these drawbacks^13–16^. The formation of spinel-like and disordered NiO rock-salt structure is regarded as the origin of voltage fade^12,17–19^. Abundant oxygen vacancies formed in the cycled Li-rich materials lead to the insertion of lithium ions into the octahedral site of defect spinel-like and disordered NiO rock-salt structure^5,12,20^. Meanwhile, the valence state of Mn ions, especially on the outmost surface, is significantly reduced to Mn^3+^ due to the Jahn–Teller distortion^5^. The Mn^3+^ on the surface is extremely unstable and easily tends to generate soluble Mn^2+^ by disproportionate decomposition, resulting in the loss of active materials and gradual decay of capacity^12,17^. The dissolution of manganese in Mn-based cathode materials seriously affects the CE and cycle stability. Furthermore, the degradation of cathode accelerated at a high operational voltage (usually operated between 2.0 and 4.8 V versus Li/Li^+^ in Li-rich). The electrolytes inevitably tend to decompose at high potential^21,22^. The result of electrolyte decomposition is the formation of cathode electrolyte interphase (CEI) layers, composed of organic components such as ROCO_2_Li, RCF*x*, and RCO_*x*_F_*y*_, polycarbonates and inorganic components such as LiF, Li_*x*_PO_*y*_F_*z*_, and Li_2_CO_3_^23,24^. The heterogeneous organic-rich CEI is found to be insulating, detrimental to the rapid transfer of electrons on the surface and facilitate the surface degradation of the cathode, accompanied by the dissolution of TM^24–26^. Zhang et al.^26^ indicated that the reconstruction of NMC811 was effectively suppressed in fluorinated orthoformate-based electrolytes due to the formation of an even and robust LiF-rich CEI on the surface of the particle.

Recently, highly concentrated electrolytes (HCEs) have been widely used in LIBs to improve cycle stability, Coulombic efficiency, and rate performance. Considerable works indicated that the anions are predominantly reduced to form an anion-derived solid electrolyte interphase (SEI) on the surface of anode, which is significantly different from the conventional solvent-derived SEI observed in the dilute electrolyte^27–33^. The anion-derived SEI exhibit not only higher stability but lower interfacial resistance compared with the solvent-derived SEI, which is beneficial to the electrochemical performance^28^. Moreover, Yamada et al.^34^ found that the dissolution of transition metals of LiNi_0.5_Mn_1.5_O_4_ was greatly inhibited via replacing the dilute electrolyte with 5.5 M LiN(SO_2_F)_2_(LiFSA)/dimethyl carbonate (DMC) electrolyte, where a peculiar 3D network of anion and solvent molecules coordinated with Li^+^ forms. At present, the discussion on the mechanism of excellent performance with concentrated electrolyte is mainly focused on the SEI film on anode side. However, the influence of interfacial chemistry between electrolyte and cathode after using highly concentrated electrolyte has not been thoroughly studied.

In our previous works^35,36^, we found that the substitution of Co with Fe in Li-rich (Fe-Li-rich) materials can inhibit the formation of peroxy bonds and phase transformation, resulting in less voltage fade and the better capacity retention. The Fe-Li-rich is becoming a competitive candidate for the next generation of cathode material for its huge cost advantage. However, we also found the low CE during cycling is extremely serious in the Fe-Li-rich cathode, and the mechanism is still indistinct.

Herein, we discuss the correlation between the nickel-iron-manganese dissolution and the low CE of the Li_1.2_Ni_0.15_Fe_0.1_Mn_0.55_O_2_ (LNFMO). We clearly elucidate that the iron dissolution ions from LNFMO is the dominant reason for the low CE rather than manganese dissolution during cycling in the conventional dilute electrolyte. On the other hand, we use the concentrated electrolyte to inhibit the dissolution and deposition of TMs. We observe an uneven and delicate organic-rich formed on the surface in the diluted electrolyte through high-resolution transmission electron microscopy (HRTEM), X-ray photoelectron spectroscopy (XPS) and time-of-flight secondary-ion mass spectrometry (TOF-SIMS), which facilitates the surface degradation of the cathode. For comparison, an inorganic-rich CEI forms in the concentrated LiPF_6_ in ethylene carbonate (EC)/ethyl methyl carbonate (EMC)/dimethyl carbonate (DMC) (1:1:1 by vol.) after long cycle period, whose highly homogeneous and robust properties greatly protect the surface of cathode from degradation, which is advantageous to the cycle stability and coulombic efficiency of the Fe-Li-rich. This work not only gives us a better understanding of the correlation between the dissolution of TM and the low CE of the Fe-Li-rich, but also provides an effective strategy to form a uniform and robust inorganic-rich CEI to inhibit the dissolution of TM in a highly concentrated electrolyte.

## Results

### Characterization of LNFMO

**Fig. 1:**
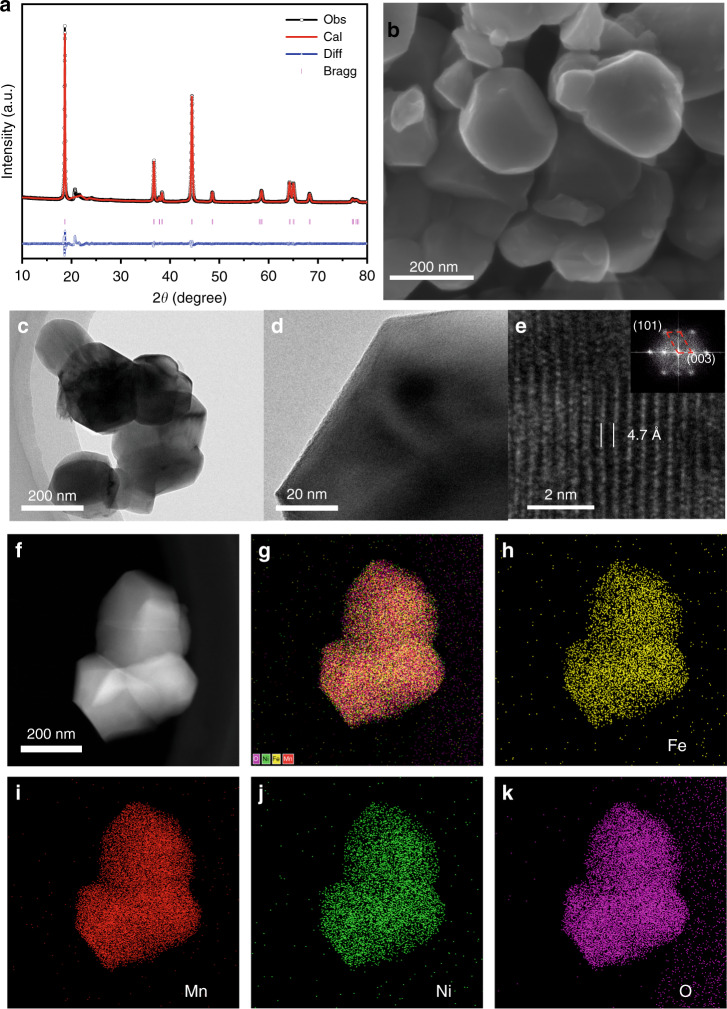
Structural characterization and EDS spectra of the LNFMO. **a** Rietveld refinement XRD pattern of LNFMO; **b** SEM image of the sample; **c**–**e** HRTEM images and corresponding FFT of the sample; **f**–**k** elemental distribution in LNFMO obtained from STEM-EDS.

We employed a sol–gel method to prepare LNFMO. The X-ray diffraction (XRD) pattern of the as-prepared LNFMO is shown in Fig. 1a. The XRD refinement of the LLNFMO was conducted by using R$\bar 3$m space group. The low intensity peaks between 20° and 23° can be assigned to the typical characteristic peaks of the monoclinic Li_2_MnO_3_-type structure with C2/m space group^35^. As expected, the XRD refinement indicates that the structure of as-prepared LNFMO is well fitted with the hexagonal α-NaFeO_2_-type structure, and the sharp and clear splitting of the (006)/(102) and (108)/(110) peaks manifest its well crystalline-layered structure. The result of Rietveld refinement is given in Supplementary Table 1. The *I*_003_/*I*_104_ intensity ratio is nearly 1.45 (>1.2), which indicates a low ratio of cation disordering^37^.

SEM images show the granular morphology of as-prepared LLNFMO with the particle size approximately 200 nm (Fig. 1b; Supplementary Fig. 1). This morphology is consistent with the observation by HRTEM (Fig. 1c). Moreover, the sharp edge and lattice fringes of LNFMO displayed interplanar spacings of 0.47 nm are clearly observed in the surface of particle (Fig. 1d, e), which matches well with the (003) plane of the LNFMO. The corresponding fast Fourier transform (FFT) of the lattice fringes also confirms the well crystalline-layered structure with R$\bar 3$m space group (Fig. 1e)^38^. The scanning transmission electron microscopy energy dispersive X-ray analysis (STEM-EDS) was employed to determine the elemental distribution of Fe, Mn, Ni and O. Obviously, the Fe, Mn, Ni, and O are uniformly distributed throughout the particles (Fig. 1f–k), which is conducive to structural stability. ICP-OES was also carried out to determine the chemical composition of the prepared LNFMO. The ICP analysis indicates that Li:Ni:Fe:Mn cation ratio is exceedingly close to the expected nominal stoichiometry (Supplementary Table 2).

### Analysis of electrolyte

**Fig. 2:**
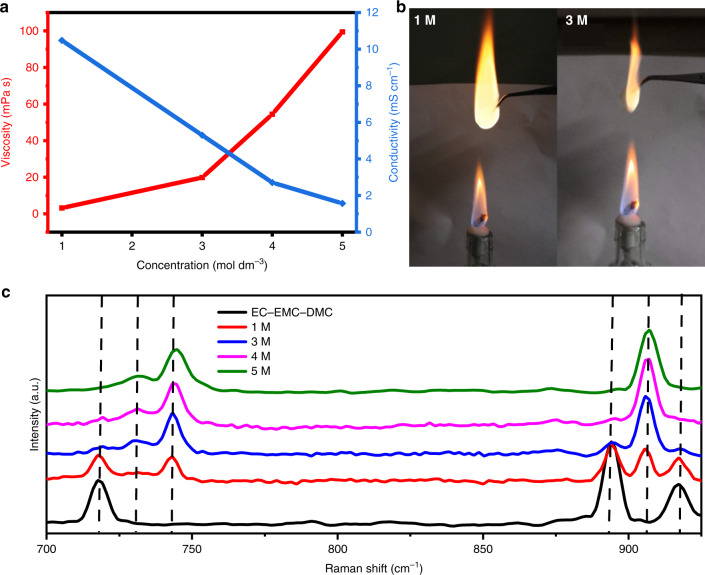
Physicochemical properties and Raman spectra dependent on salt concentration. **a** Viscosity and ionic conductivity for electrolyte of LiPF_6_ in EC:EMC:DMC (1:1:1 in vol.) at 25 °C; **b** flame tests of a dilute electrolyte of 1 mol dm^−3^ LiPF_6_/EC:EMC:DMC and a concentrated electrolyte of 3 mol dm^−3^ LiPF_6_ /EC:EMC:DMC; **c** Raman spectra of EC:EMC:DMC and various salt concentration electrolyte.

Electrolytes with various concentrations were prepared by dissolving different stoichiometric ratios of LiPF_6_ salt in the mixed carbonate ester solvents (EC:EMC:DMC = 1:1:1 by vol.). Their detailed basic physicochemical properties are presented in Supplementary Table 3. Figure 2a shows the relationships of viscosity and ionic conductivity with salt concentration. The viscosity increases significantly with the increase of salt concentration from 1 to 5 M, whereas the corresponding ionic conductivity decreases sharply. Flammability is the crucial property of electrolyte that profoundly affects battery safety^39^. Flame tests of lab-made concentrated electrolyte and dilute commercial electrolyte are present in Fig. 2b, which shows the higher flammability of dilute commercial electrolyte (1 M) than that of concentrated electrolyte (3 M). Raman spectroscopy was applied to characterize the structure of the various electrolytes (Fig. 2c). Three major bonding modes approximately at 717, 894, and 917 cm^−1^ are assigned to the vibration of free carbonate-based solvent (black line). The peak at ~743 cm^−1^ corresponds to the symmetric vibration of the LiPF_6_ (PF_6_^−^) in the solvent, whose intensity increases with the increase of concentration^40^. Similarly, the intensity of peak at ~905 cm^−1^ increases remarkably with increasing salt concentration, which indicates this bonding mode belongs to the coordinated solvent with Li^+^. Particularly, the peaks of the free solvent gradually disappear as the salt concentration increases, which demonstrate that the free solvent molecules disappear gradually. At the same time, a new peak at ~731 cm^−1^ appears when the salt concentration exceeds 3 M, which might signifies the formation of aggregates (AGGs, a PF_6_^−^ coordinating to two or more Li^+^)^28,34^. As a result, a unique solution structure is obtained in concentrated electrolyte^28,41,42^.

### Electrochemical performance and the dissolution of TM

**Fig. 3:**
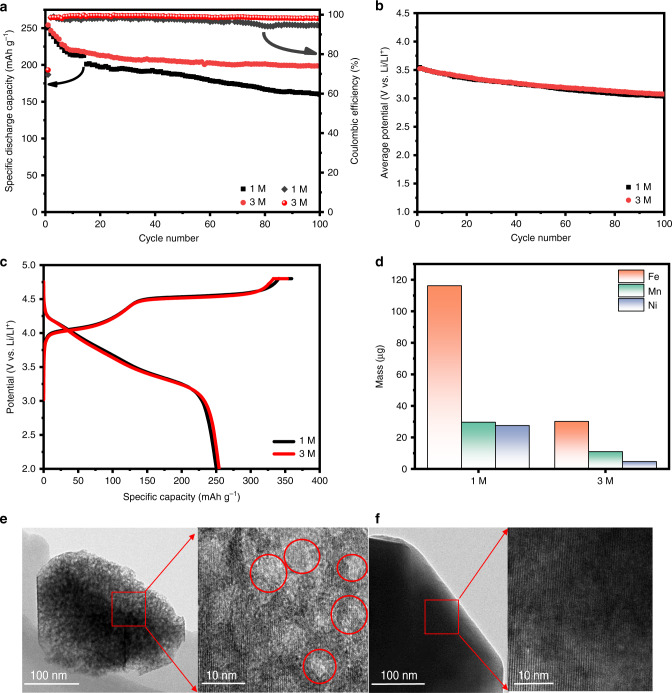
Electrochemical performance of LNFMO and analysis of the dissolution of TM. **a** Cycling performance in 1 and 3 M electrolytes at 0.1 C (20 mA g^−1^); **b** the dependence of average discharge potential on cycle number; **c** the initial charge-discharge curve at 0.1 C in 1 and 3 M electrolyte; **d** the amount of transition metal deposited on the 100 cycled lithium plate in 1 and 3 M electrolytes; **e**, **f** the HRTEM images of 100 cycled electrodes in 1 and 3 M electrolytes, respectively.

Our previous works indicated that voltage fade was significantly suppressed by replacing Co with Fe^35,36^. Unfortunately, the low coulombic efficiency during cycling has always existed but is rarely reported in Fe-substituted Li-rich cathode materials^20,43–45^, which inevitably gives rise to poor cyclic performance and reduces battery life. In order to intuitively investigate the mechanism of low CE of LNFMO, the LNFMO electrodes were cycled in different concentrations of electrolyte at 0.1 C (20 mA g^−1^). In dilute electrolyte (1 M LiPF_6_/EC-EMC-DMC), the CE remains around 97% before 75 cycles, and drop to only about 94% at the end of the cycle (Fig. 3a), indicating irreversible decay of the structure and continuous depletion of the electrolyte due to the poor protection function of the cathode electrolyte interface^24^. As the LiPF_6_ concentration increases from 1 to 3 M, the CE immediately increases from 97% to around 99.2% with no significant decrease at the end of the cycle. Besides, the LNFMO delivers a higher initial discharge capacity and a higher initial CE of 72.5% in 3 M electrolyte sharply contrast to 69% in 1 M electrolyte (Fig. 3a, c), which might be plausibly explained by reducing the dissolution and migration of TM as well as oxygen activity of the LNFMO in concentrated electrolyte^14^. Notably, the electrode cycled in 3 M electrolyte shows the excellent cycle stability with 200 mAh g^−1^ (79% of capacity retention) after 100 cycles, comparing to the electrode cycled in 1 M electrolyte with only 160 mAh g^−1^ (64% of capacity retention). The electrode cycled in 1 M electrolyte exhibits lower average potential, which is noticeably enhanced in 3 M electrolyte (Fig. 3b). Average voltage increased by ~50 mV after long cycle in 3 M electrolyte compared to 1 M electrolyte (Supplementary Fig. 2).

So as to investigate the mechanism of poor stability and low CE of LNFMO, various post-mortem analysis techniques were carried out. It is well known that the dissolution of TM is the main responsibility of poor cycle stability and low CE of cathode materials, especially operated at high voltage (>4.5 V)^15,16^. TEM images of electrodes extracted from cells cycled 100 cycles at 0.1C in 1 and 3 M electrolytes are shown in Fig. 3e, f. Obviously, the electrode in 1 M electrolyte is attacked violently by HF^16^ (Fig. 3e) and the lattice fringes of electrode undergo severe corrosion (red circles). As a result, some soluble fragments, such as MnF_*n*_, NiF_*n*_, FeF_*n*_ etc., are formed on the outside surface and then dissolved into the electrolyte^23^. Nevertheless, the corrosion attacked by HF is inhibited markedly in 3 M electrolyte for the formation of passivation films on the surface of electrode (Fig. 3f). SEM-EDS images of corresponding lithium counter electrodes manifest the different degrees of deposition of Mn, Fe, and Ni on Li metal (Supplementary Fig. 3). The analysis results of deposition of each TM elements with ICP-OES indicate that the amount of iron deposition is the highest far beyond other TM elements (Fig. 3d). Although the deposition of TM, especially Fe, on the Li metal electrode is markedly inhibited in 3 M electrolyte, where some soluble species including MF_*n*_ are hardly dissolved^34^ due to the unique a 3D network solution structure of AGGs-predominant concentrated electrolyte. The dissolution of Fe rather than Mn is the primary cause for poor cycle stability and low CE of the LNFMO, leading to a better understanding of the mechanism of poor stability and low CE of Co-free Li-rich cathode materials.

**Fig. 4:**
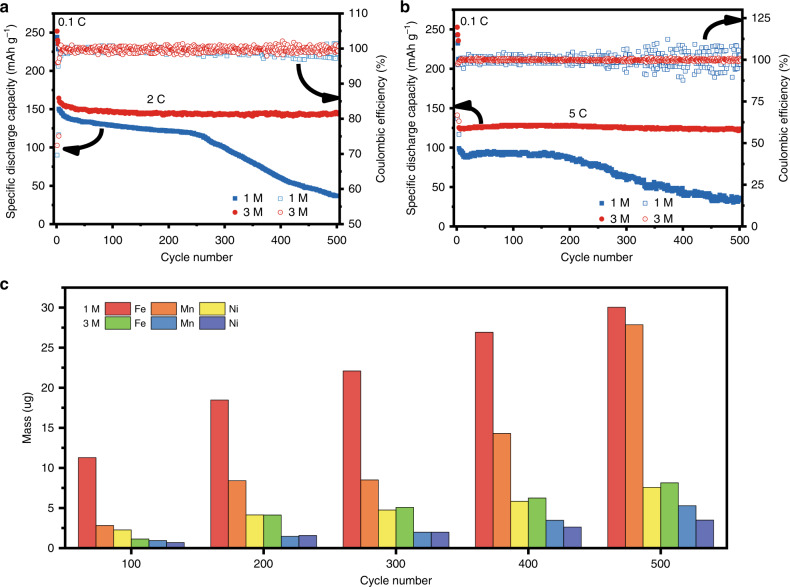
Rate performance of electrodes at 25 °C and analysis of the deposition of TM. **a**, **b** The cycle performance in 1 and 3 M electrolyte at 2C and 5C, respectively; **c** the amount of transition metal deposited on the different cycled lithium plates in 1 and 3 M electrolytes at 2C.

**Fig. 5:**
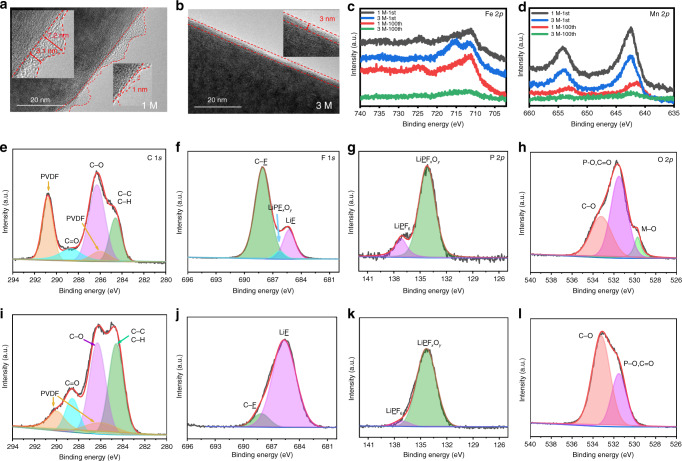
Electrode passivation in 1 and 3 M electrolytes. **a**, **b** HRTEM images of electrodes after cycling in 1 and 3 M electrolyte, respectively; **c**, **d** XPS spectra of Fe 2*p* and Mn 2*p* on the electrodes cycled in 1 and 3 M electrolyte after 100 cycles at 0.1C, respectively; **e**–**h** C 1*s*, F 1*s*, P 2*p*, and O 2*p* spectra on the cycled electrode in 1 M electrolyte; **i**–**l** C 1*s*, F 1*s*, P 2*p*, and O 2*p* spectra on the cycled electrode in 3 M electrolyte.

Rate performance of the LNFMO cycled in various different concentrations has been investigated (charged and discharged at the same rate). As shown in Supplementary Fig. 4, the reversible discharge capacity of LNFMO in traditional dilute electrolyte decreases sharply as the rate increases. For comparison, all the concentrated electrolytes exhibit higher reversible discharge capacity at all different C-rate. This can be attributed to a unique Li^+^-conduction mechanism in concentrated electrolyte^28^. Figure 4a shows the cycling stability of electrode in 1 and 3 M electrolyte at 2C rate. The electrode in 3 M electrolyte exhibits excellent cyclic stability with 150 mAh g^−1^ (94% capacity retention) after 500 cycles. Unfortunately, the electrode in 1 M electrolyte exhibits terrible cyclic stability with 25 mAh g^−1^ (17% capacity retention) after 500 cycles. These are also happened when cycled at 5 C rate (Fig. 4b). It is worth mentioning that the electrodes in concentrated electrolyte exhibit excellent cycle stability with 125 mAh g^−1^ (almost 100% capacity retention) after 500 cycles and almost 100% coulombic efficiency. In contrast, the discharge capacity decreases rapidly after approximately 250 cycles. We measured the number of deposited transition metal after different cycles (Fig. 4c). It is clearly shown that the deposited quantity of TM in 1 M electrolyte increases markedly with cycling. Notably, the dissolution and deposition of TM are tremendously suppressed in 3 M electrolyte. The deintercalation of lithium ions at high rates will accelerate the dissolution of TMs, especially in 1 M electrolyte (Supplementary Fig. 5). A large number of TM ions are irreversibly detached from the material, reducing the CE, and then react with the electrolyte to accelerate the aging of the cells. Fortunately, the discharge capacity decay and voltage fade are validly suppressed in 3 M electrolyte at high rate (Supplementary Fig. 6), indicating the concentrated electrolyte can stabilize the structure of electrode. Electrochemical impedance spectroscopy was conducted to explain the high rate performance in concentrated electrolyte (Supplementary Fig. 7). As the number of cycles increases, the semicircle at low frequency, including the resistance of charge transfer and CEI, gradually expands in 1 M electrolyte but remains substantially unchanged in 3 M electrolyte, indicating that the stability of CEI on the electrode in 3 M electrolyte is higher than that in 1 M electrolyte. Raman spectra were carried out to investigate the change of LNFMO structure (Supplementary Fig. 8). Two sharp peaks near 480 and 595 cm^−1^ of the pristine LNFMO are assigned to Eg and A1g vibration modes for the symmetrical deformation and symmetrical stretching of metal-oxygen, respectively^46,47^. The spinel phase will be inevitably formed after long-term cycles due to the phase transformation, which manifests a sharp A1g peak at 630 cm^−1^^48^. The A1g peak at around 600 cm^−1^ does not shift obviously to 630 cm^−1^, which indicates the phase transformation is greatly suppressed in the Fe-substituted Li-rich cathode materials. In addition, the proportion of the peak at 630 cm^−1^ representing the spinel phase is significantly smaller in 3 M electrolyte, accounting for the valid suppression of discharge capacity decay and voltage decay in 3 M electrolyte. Supplementary Figure 9 displays the excellent cyclic stability at 2C rate even at 40 °C in concentrated electrolyte, where the capacity retention is increased to 75% compared with 49% in 1 M electrolyte. To our delight, a robust (3 M) rather than friable (1 M) passivation film formed on the surface in concentrated electrolyte to protect the particles from being attacked constantly by HF (Fig. 5a, b; Supplementary Fig. 10). The fluorine content on the surface of electrode is 2.3 times that of the original in 3 M electrolyte compared with the 1.4 times in 1 M electrolyte (Supplementary Figs. 11 and 12). The same is true for the change in phosphorus content. All these results manifest that an inorganic-rich CEI (LiF-rich) formed rather than an organic-rich CEI in concentrated electrolyte, which is beneficial to preventing the cathode from declining.

### Formation of CEI

The significant improvement of cycle stability and rate performance of the concentrated 3 M LiPF_6_/EC-EMC-DMC compared with dilute 1 M electrolyte can be explained powerfully by the distinction of CEIs generated in two different electrolytes. A fragile and inhomogenous CEI layer is clearly observed on the electrode cycled in 1 M electrolyte (Fig. 5a). Apparently, the thickness of the CEI layer is extremely uneven. The thickest is nearly 7.2 nm and the thinnest is only 1 nm, which facilitates the surface degradation of cathode^21^. Although a robust and uniform CEI layer generated on the electrode cycled in 3 M electrolyte (Fig. 5b), whose thickness remain uniform (around 3 nm), protecting the electrode from degradation. Fe and Mn are not detected on the outmost surface of electrode cycled in 3 M electrolyte, confirmed by the XPS spectra of Fe 2*p* and Mn 2*p* on the electrodes cycled in 1 and 3 M electrolyte after 100 cycles at 0.1C (Fig. 5c, d), owing to the robust and dense uniform LiF-rich CEI layer formed on the outmost surface of electrode^26^. In comparison, Fe and Mn are detected on the surface of electrode cycled in 1 M electrolyte due to the uneven and fragile organic-rich CEI layer generated on the outmost surface of electrode. More detailed analysis of the CEI layer on the surface of electrode cycled in 1 and 3 M electrolyte are shown in Fig. 5e–l. The XPS spectra of C 1*s* (Fig. 5e–i) is considered to be the representative of the organic-rich CEI, derived from the carbonate solvents, generally including C–C/C–H, C–O and C=O^26^. The intensity ratio of C–O/C–C in 3 M electrolyte is much larger in 1 M electrolyte, signifying more organic species (e.g., RCO_*x*_Li) formed in 1 M electrolyte. These organic species are identified as fragile and unstable. The intensity of C-F (PVDF) in Fig. 5e is higher than in Fig. 5i, whereas M-O (529.8 eV)^49^ exists in Fig. 5h and no M-O exists in Fig. 5l, indicating the inorganic-rich CEI formed in 3 M electrolyte is extremely robust. The XPS spectra of F 1*s* and P 2*p* are considered to be the representative of the inorganic-rich CEI (Fig. 5f–j, g–k)^26,50^, derived from the LiPF_6_ salt. A small amount of LiF exists in the CEI (organic-rich) formed in 1 M (Fig. 5f). In contrast, the only existence of LiF (Fig. 5j) indicated the CEI layer formed in concentrated electrolyte is a LiF-rich CEI^26^. The XPS spectra of P 2*p* also indicates that there are more inorganic species (e.g., LiPO_*x*_F_*y*_) in CEI formed in 3 M electrolyte. In addition, the atomic ratio of C, O, F and P on the surface of CEI indicates that the Li-rich CEI is preferentially formed in the concentrated electrolyte (Supplementary Fig. 13). All the results indicate that LiPF_6_ salts are prefer to decomposing and participating in the CEI formation in concentrated electrolyte, which is extraordinarily different from the carbonate solvent preferentially involved in the CEI formation in dilute electrolyte. The LiF-rich CEI layer will effectively passivate the electrode surface, thereby inhibiting the continuous dissolution of the transition metal and bringing better thermal stability (Supplementary Fig. 14).

**Fig. 6:**
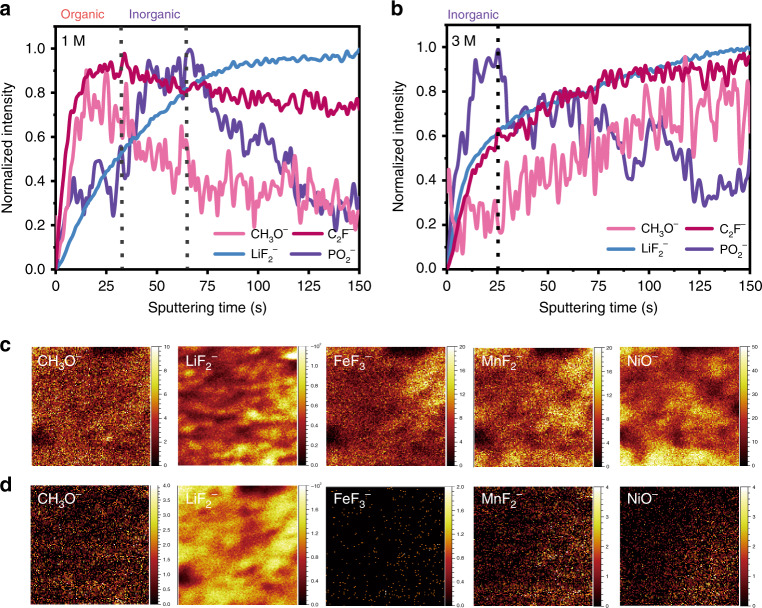
TOF-SIMS spectra of interphases on the surface of cycled electrodes. **a**, **b** Normalized (to maximum) depth profiling of several typical second ion fragments on the cycled electrode outside surface; **c**, **d** TOF-SIMS chemical maps of several typical second ion fragments with 150 s of sputtering on electrodes cycled in 1 and 3 M electrolyte, respectively.

In order to provide direct evidence of the CEI layer formed in 1 and 3 M electrolyte, TOF-SIMS was carried out to characterize the surface chemical composition (Fig. 6). Plenty of secondary-ion fragments (e.g., CH_3_O^−^, C_2_HO^−^, C_3_O_2_F^−^, C_2_F^−^, LiF_2_^−^, PO_2_^−^, FeF_2_^−^, FeF_3_^−^, MnF_2_^−^, NiF_3_^−^, and NiO^−^) are obtained, which are a function of sputtering time. The CH_3_O^−^, C_3_O_2_F^−^, and C_2_F^−^ fragments are generally considered as the organic species of CEI; the LiF_2_^−^ and PO_2_^−^ fragments are usually regarded as the inorganic species of CEI; and the FeF_2_^−^, FeF_3_^−^, MnF_2_^−^, and NiF_3_^−^ fragments commonly belong to the dissolved product of transition metals on the surface^16,21,23^. The normalized TOF-SIMS depth profiles of representative CEI signals are shown in Fig. 6a, b. The maximum normalized intensity of CH_3_O^−^ and C_2_F^−^ occur earlier than PO_2_^−^ (Fig. 6a), indicating the outer layer is mainly fragile organic species in 1 M electrolyte. On the contrary, the signals of LiF_2_^−^ and PO_2_^−^ are obtained earlier than CH_3_O^−^ and C_2_F^−^ in 3 M electrolyte, indicating the outer layer is mainly robust inorganic species^23^. Visual evidence is presented in Supplementary Fig. 15, where there are more organic species (CH_3_O^−^, C_2_HO^−^, C_3_O_2_F^−^, C_2_F^−^) in the outer layer in 1 M electrolyte compared with more inorganic species (LiF_2_^−^, PO_2_^−^) in the outer layer in 3 M electrolyte. In addition, the number of organic species (CH_3_O^−^), inorganic species (LiF_2_^−^) and MFx (FeF_3_^−^, MnF_2_^−^) as well as rock-salt NiO (NiO^−^) in the organic-rich CEI layer are in sharp contrast with the inorganic-rich CEI layer (Fig. 6c, d). The corresponding depth profiles of several fragments also reveal that the intensity of organic species, dissolution products (MF_*x*_, M = Mn, Fe), and NiO^−^ on the electrode surface cycled in 1 M electrolyte are far higher than in 3 M and the intensity of inorganic species (LiF_2_^−^) is reversed (Supplementary Figs. 16 and 17). These results are consistent with the previous XPS and SEM-EDS analysis. In addition, the higher intensity of FeF_2_^−^, FeF_3_^−^, MnF_2_^−^, and NiF_3_^−^ fragments on the electrode surface cycled in 1 M electrolyte are presented clearly, manifesting visually a large amount of transition metal dissolved on the surface^25^. However, only a very small amount of the transition metal dissolves on the surface in 3 M electrolyte. All of these results exhibit direct evidence for the inhibition of transition metal dissolution mechanism in high salt concentration electrolytes. The less rock-salt NiO formed on the electrode surface cycled in 3 M electrolyte, indicating that phase transition is effectively suppressed in concentrated electrolyte^17,23^.

## Discussion

**Fig. 7:**
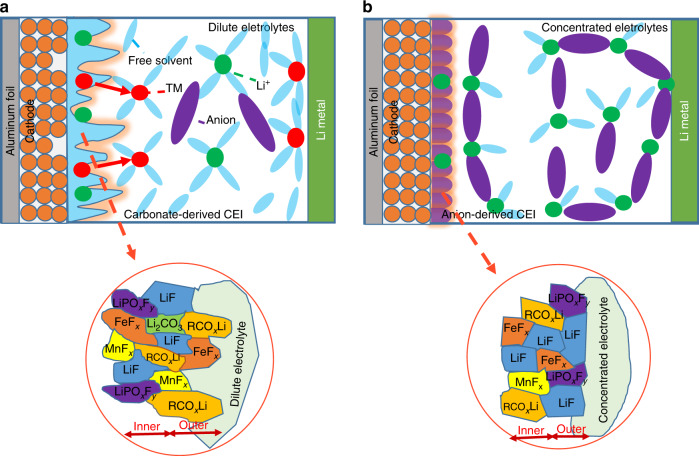
Schematic illustrations of passivation films in 1 and 3 M electrolytes. **a** Schematic of carbonate-derived (organic-rich) CEI formed on the surface in 1 M electrolyte; **b** schematic of inorganic-rich CEI formed on the surface in 3 M electrolyte.

The dissolution of transition metals, especially iron, seriously affects the performance of Li_1.2_Ni_0.15_Fe_0.1_Mn_0.55_O_2_, whereas the CEI layer formed on the surface also has a profound effect on the dissolution of transition metals. Conclusively, the CEI layer has a direct relationship with cycling stability, coulombic efficiency, rate performance, and safety. From the above results, the excellent performance of LNFMO in concentrated electrolytes includes cycling stability, rate performance, CE, and safety owing to the formation of (LiF-rich) CEI. Corresponding schematic illustrations of passivation films generated in 1 and 3 M electrolytes are shown in Fig. 7. A fragile and uneven carbonate-derived CEI (organic-rich), formed in dilute electrolyte upon cycling (Fig. 7a), failed to prevent the cathode from being attacked by HF. This phenomenon results in the number of dissolved products (MF_*x*_, such as MnF_*x*_, FeF_*x*_). These dissolved products are generated on the surface, coordinated with free solvents, then deposited in the Li metal electrode via the ion-exchange process^15^, which causes the degradation of cycling performance, low CE, and poor rate performance. For comparison, a unique solution structure is obtained in concentrated electrolyte. As a result, the dissolved products are difficult to coordinate with solvents. Furthermore, the uniform and robust LiF-rich CEI formed on the surface effectively protect materials from attack by HF, leading to less dissolved products observed on the surface (Figs. 5c, d and Fig. 6g–i). This provides a piece of direct evidence to explain the excellent cycle durability and high CE cycled in 3 M electrolyte. The excellent rate performance is due to the high ionic transport properties (e.g., unique Li^+^-conduction mechanism). The schematic diagrams of the chemical composition of CEI layer on the surface of electrodes are roughly shown based on TOF-SIMS analysis. The CEI layer is supposed to be a complicated multilayer structure. There are obvious differences between the carbonate-derived and LiF-rich CEI. The interface between the carbonate-derived CEI and dilute electrolyte is rugged, where the outer layer is mainly composed of organic species (e.g., RCO_*x*_Li) and some dissolved products. While, the interface between the LiF-rich CEI and concentrated electrolyte is flat and the outer layer is mainly composed of inorganic species (e.g., LiF). Our finding of the chemical composition of CEI layer provides a better understanding of the complicated CEI layer and more specific components still need to be further investigated.

In summary, we elucidate the definite correlation between the nickel-iron-manganese dissolution and the low CE of the LNFMO. The Fe dissolution is more serious than Mn dissolution in the LNFMO, which not only accelerates the capacity decay but also significantly reduces the CE. Increasing salt concentration can markedly improve the cycling stability, CE, rate performance, and safety of the LNFMO. We conduct in-depth discussions on the mechanism of the significant improvement in concentrated electrolyte. The transition metals dissolution of cathode is markedly inhibited in concentrated electrolyte due to the LiF-rich CEI formed on the surface upon cycling. The LiF-rich CEI is so uniform and robust to effectively protect the cathode materials from erosion. We demonstrate the direct evidence of the difference between the carbonate-derived and LiF-rich CEI layer. The outer layer of carbonate-derived CEI is mainly organic species and dissolved products, which is sharp different from the inorganic species in LiF-rich CEI. This work provides an effective strategy to inhibit the dissolution of transition metals of the Co-free Li-rich cathode materials.

## Methods

### Material preparation

The Li_1.2_Ni_0.15_Fe_0.1_Mn_0.55_O_2_ (LNFMO) was synthesized via the sol–gel method. Stoichiometric amounts of the mixture of Ni(CH_3_COO)_2_·4H_2_O (99%, Aladdin), Fe(NO_3_)_3_·9H_2_O (99%, J&K Chemical), Mn(CH_3_COO)_2_·4H_2_O (99%, Aladdin), and CH_3_COOLi·2H_2_O (5 at% excess, 99%, Macklin) were dissolved into deionized water, which was injected dropwise into an appropriate amount of citric acid (99%, inno-chem) solution to obtain a clear green solution. Afterward, the pH value of solution was adjusted to neutral using aqueous ammonia. The gel was obtained by heating the solution at 85 °C with vigorous stirring for 6 h and then heated at 200 °C in oven to eliminate citric acid. After that, the obtained fluffy porous black precursor was calcined at 450 °C for 5 h and then at 900 °C for 24 h in air.

### Preparation of electrolytes and electrodes

Different concentrations of electrolytes were prepared by adding the corresponding amount of LiPF_6_ into the mixed solvents of EC-EMC-DMC (1:1:1 by vol.) in an argon-filled glovebox. All of the above salts, solvents and the traditional diluted 1 M electrolytes were purchased from Suzhou Duoduo Chemical Technology Co., Ltd. The electrodes were prepared via ball milling the LNFMO with polyvinylidene difluoride (PVDF) and Super P conductive carbon in *N*-methyl pyrrolidone (NMP) at a weigh on the Al foil and then dried at 110 °C under vacuum for 24 h. The electrodes with an ht ratio of 8:1:1. The obtained slurry was cast active mass loading of 2–2.5 mg cm^−2^ and a fresh Li plate were prepared for use in half- cells.

### Electrochemical measurements

The half-cells of LNFMO|Li were assembled in 2025-type coin cell in an Argon-filled glovebox. A combined separator with the glass fiber (Whatman HF/D) placed on the counter electrode side and the Celgard 2400 separator placed on the work electrode side was used in half-cells. The volume of coin cells was 180 μl to fully wet the glass fiber separator. The coin cells were tested at different C rate (1C = 200 mA g^−1^) with galvanostatic charge-discharge in the range of 4.8–2.0 V by using NEWARE-BTS. A constant voltage mode was adopted at the end of the charge, three-electrode cell (EL-CELL) was adopted for the electrochemical impedance spectra (EIS) measurements on PARSTAT2273 (AMEITEK). EIS measurements were conducted with 5 mV amplitude from 0.1 Hz to 100 KHz after being constant-current discharged to 50% state of charge of cell. All the tests were done at 25 °C except for specific high temperatures.

### Characterization

The XRD pattern was collected on Bruker D8 Advance between 10° and 80° (2*θ*) employing Cu Kα radiation, and the refined pattern was acquired by Fullprof program with the Rietveld method. SEM (Zeiss Merlin) and HRTEM (JEM 2100F) were adopted to characterize the morphology and elemental distribution of pristine LLNFM and cycled electrodes. The actual chemical composition of the prepared LNFMO was determined by ICP-OES (Thermos IRIS Intrepid II). All of the lithium plates (*d* = 13 mm) were transferred to measure the amount of deposited transition metal via ICP-OES. The viscosity and conductivity of electrolytes were acquired from Rheometer (Aaton Paar Physica MCR301) and Conductivity meter (Mettler Toledo S230), respectively. The Raman analysis was conducted using HORIBA Evolution with 532 nm laser. XPS was conducted on the long cycled electrodes using Thermo ESCALAB 250XI with monochromatic Al Kα radiation and Mg/Al double anodes light sources. The thermal stability analysis was conducted employing differential scanning calorimetry (DSC, Mettler Toledo DSC1) using gold-plated high-pressure crucible. TOF-SIMS was applied to accurately characterize the chemical composition of CEI formed on the surface of the cycled electrode via TOF-SIMS5 (ION-TOF-GmbH). A pulsed 30 KeV Bi^+^ ion beam was set, and the selected analysis area was 100 × 100 um. Then the 500 eV Cs^+^ ion beam with the incident angle of 45° (sputtering rate was 0.05 nm s^−1^ for SiO_2_) was applied to sputtering the cycled electrodes. During the analyses of the cycled electrodes, all of the samples were immersed in DMC for 24 h and dried in an argon-filled glovebox.

## Supplementary information

Supplementary Information

Peer Review File

## Data Availability

The authors declare that the data supporting the findings of this study are available within the paper and its Supplementary Information file.
