# Supplementary material for: Inhibition of transition metals dissolution in cobalt-free cathode with ultrathin robust interphase in concentrated electrolyte
Source: Nat Commun. 2020 Jul 20;11:3629. doi: 10.1038/s41467-020-17396-x (PMC7371675; doi:10.1038/s41467-020-17396-x)
Supplement: Supplementary file 1 — Supplementary Information [file 41467_2020_17396_MOESM1_ESM.pdf]

Supplementary information

Inhibition of transition metals dissolution in cobalt-free cathode with ultrathin robust interphase in concentrated electrolyte

Liu *et al.*

Supplementary Table 1. Structure parameters of $Li_{1.2}Ni_{0.15}Fe_{0.1}Mn_{0.55}O_2$, space group： $R\bar{3}m$

Sample	Lattice parameters			v（$Å^3$）	I_{003}/I_{104}	Rp(%)	Rwp（%）
	a（Å）	c（Å）	c/a				
$Li_{1.2}Ni_{0.15}Fe_{0.1}Mn_{0.55}O_2$	2.8676	14.2760	4.9784	101.6654	1.45	7.61	8.73

Supplementary Table 2. **Chemical compositions acquired from ICP-OES analysis**

Sample	Theoretical molar content of Li:Ni:Fe:Mn	Experimental molar content of Li:Ni:Fe:Mn
$Li_{1.2}Ni_{0.15}Fe_{0.1}Mn_{0.55}O_2$	1.20:0.15:0.10:0.55	1.199:0.159:0.100:0.542

Supplementary Table 3. Physicochemical Properties of electrolytes at 25°C

Concentration ($mol \cdot dm^{-3}$)	Density ($g \cdot cm^{-3}$)	Viscosity ($mPa \cdot s$)	Conductivity ($mS \cdot cm^{-1}$)
1	1.188	3.187	10.48
3	1.327	19.809	5.30
4	1.432	54.434	2.71
5	1.509	99.471	1.57

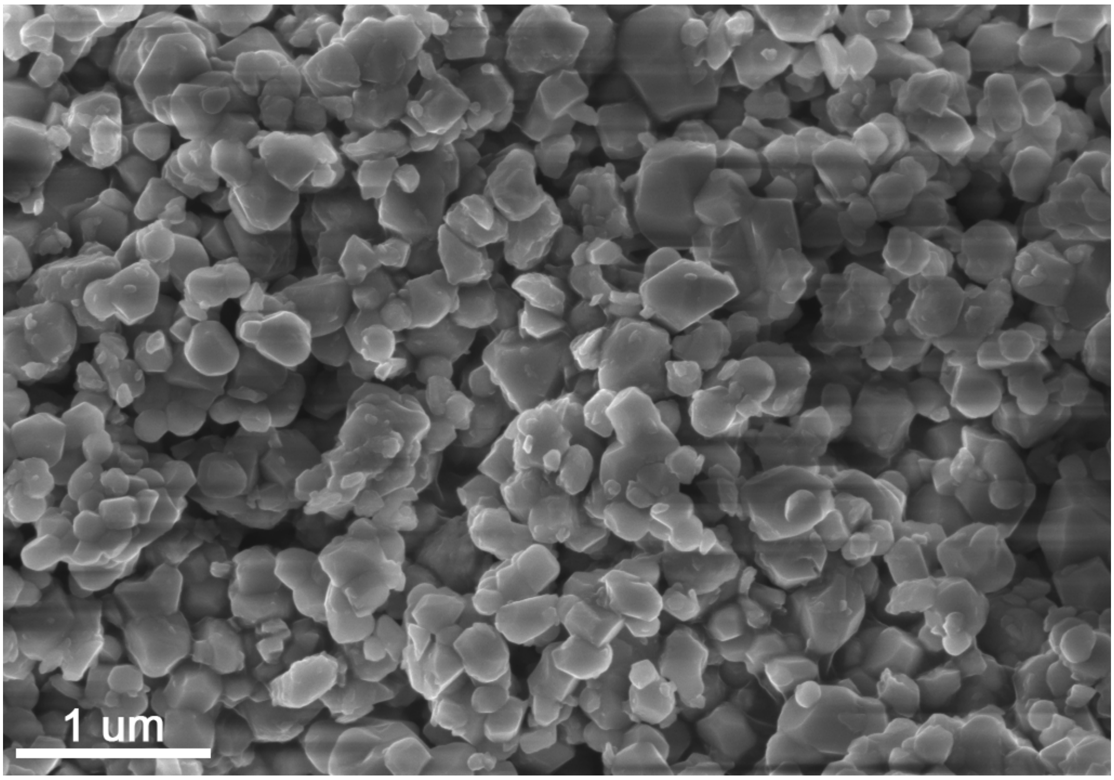

Supplementary Fig. 1. SEM image of pristine sample.

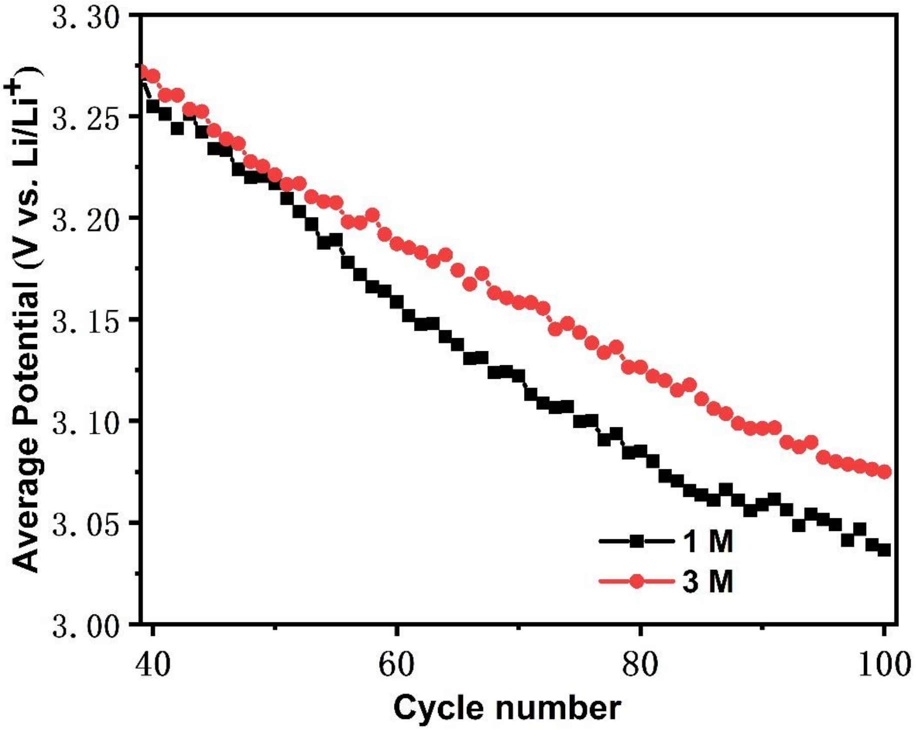

Supplementary Fig. 2. The corresponding zoom of Fig. 3b.

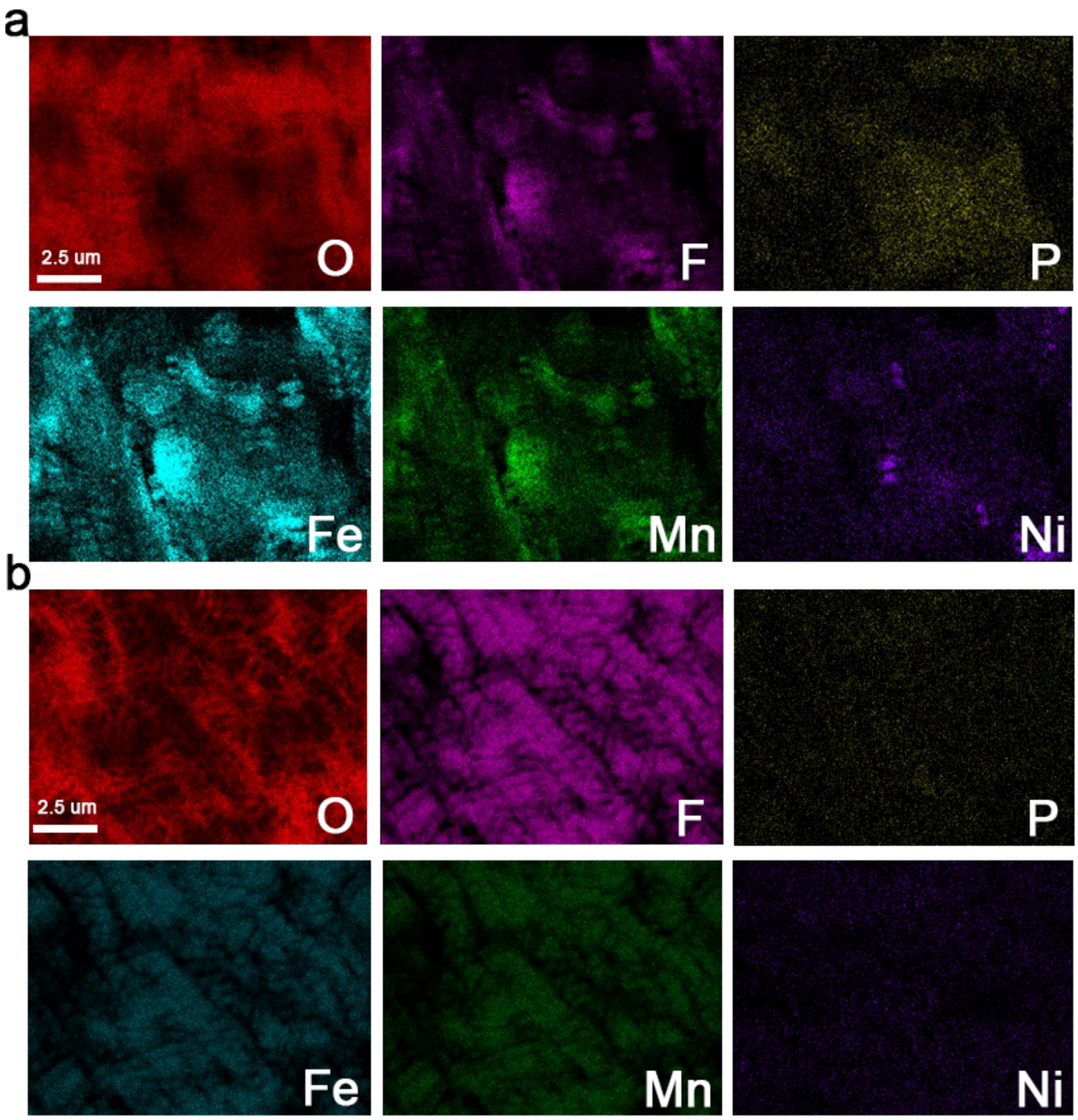

Supplementary Fig.3. The SEM-EDS spectra of 100 cycled lithium counter electrode. The SEM-EDS spectra of Lithium counter electrode cycled 100 cycles in 1 M electrolyte (a), and 3 M electrolyte (b), respectively.

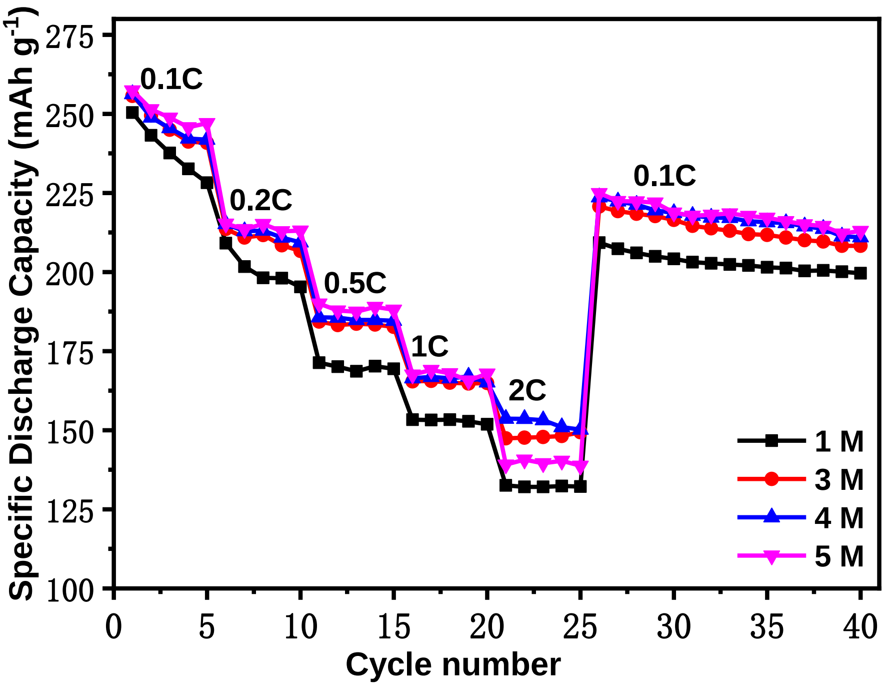

Supplementary Fig.4. Specific discharge capacity in various electrolytes at various C-rates

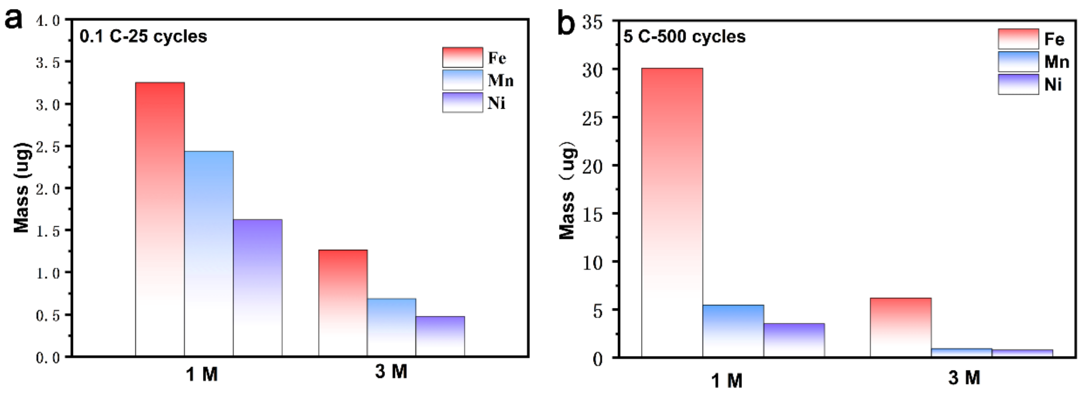

Supplementary Fig. 5. The amount of deposited transition metals on lithium metal. The amount of transition metals deposited on lithium metal counter electrode after 25 cycles at 0.1 C（a） and 500 cycles at 5 C (b), respectively.

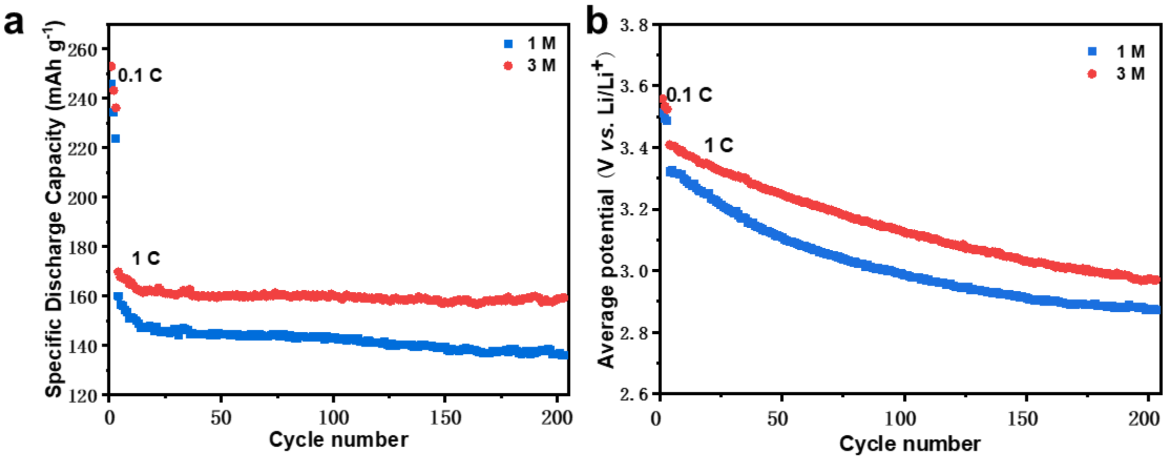

Supplementary Fig. 6. The electrochemical performance of LNFMO at 1 C. (a) The cycle performance at 1C in 1M and 3M electrolyte at 25 °C. (b) The corresponding average discharge potential at 1C in 1M and 3M electrolyte at 25 °C.

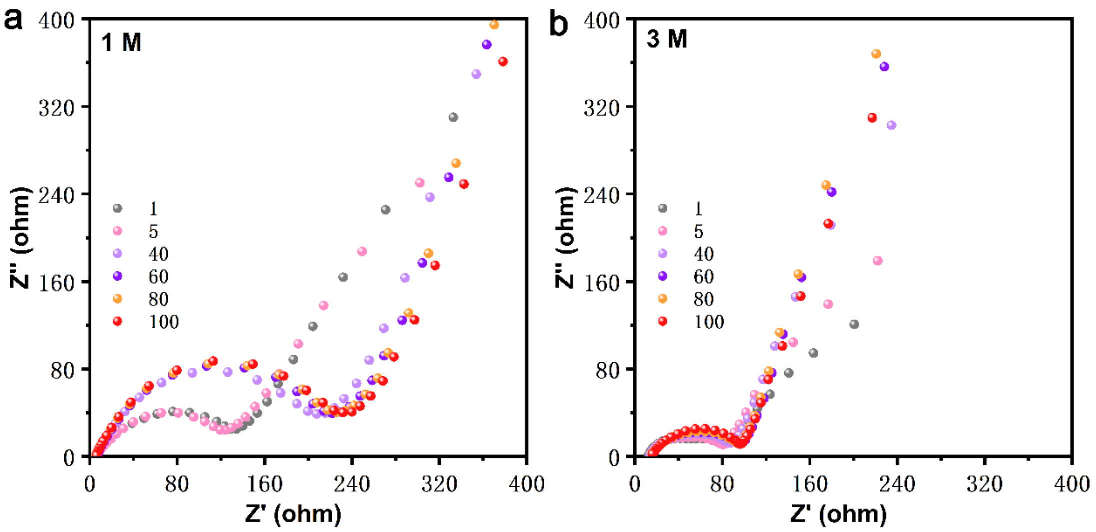

Supplementary Fig. 7. The dependence of EIS spectra of electrode with cycle number. The dependence of EIS spectra of LNFMO electrode discharged to the 50% state of charge at 1 M electrolyte (a) and 3 M electrolyte (b) with cycle number.

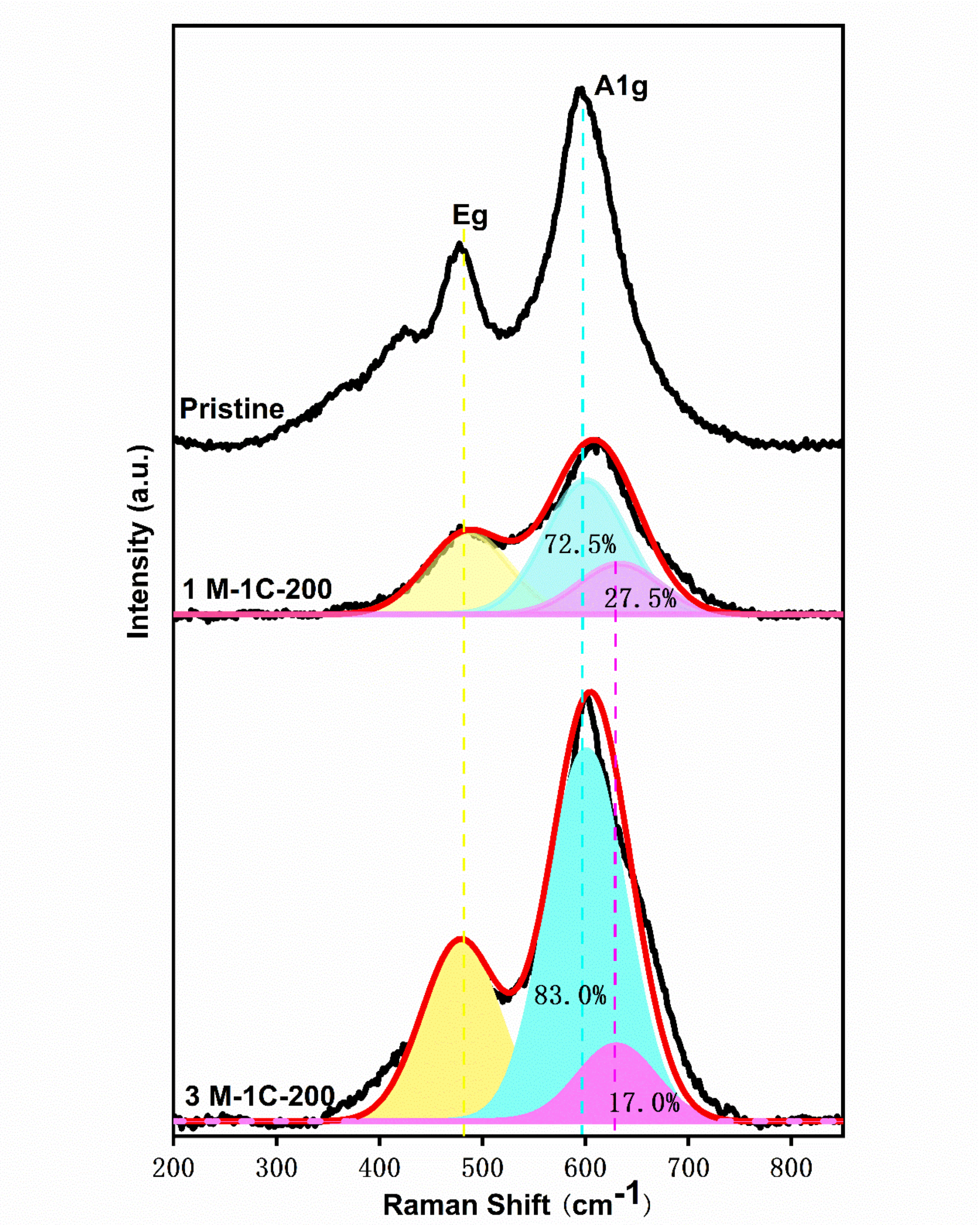

Supplementary Fig. 8. The Raman spectra of pristine LLNFMO and the electrodes after 200 cycles at 1C in 1 M and 3 M electrolyte.

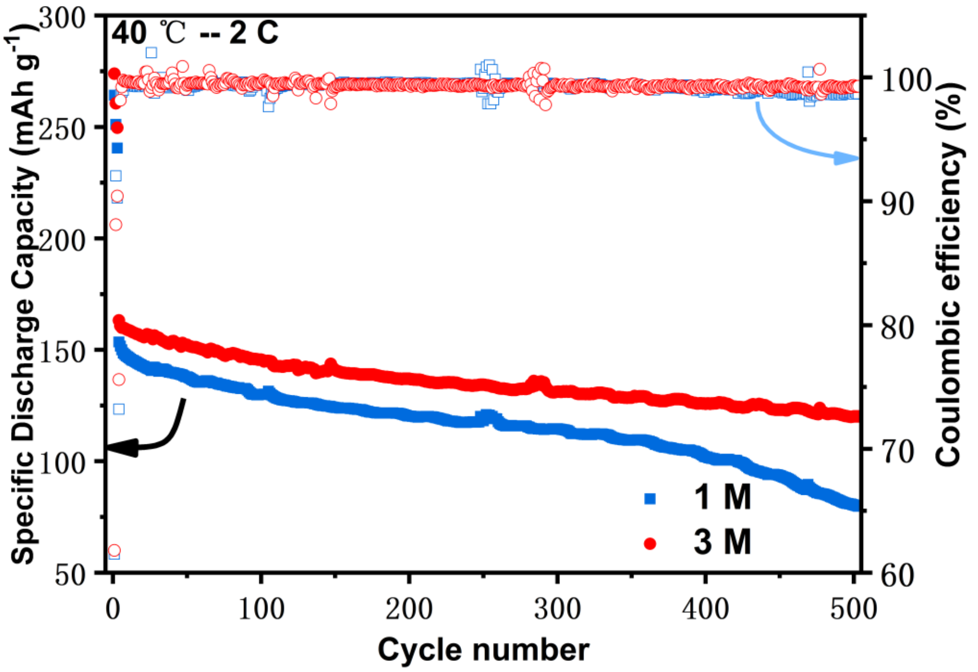

Supplementary Fig.9. The 2 C rate cycle performance in 1 M and 3 M electrolytes at elevated temperature (40 °C).

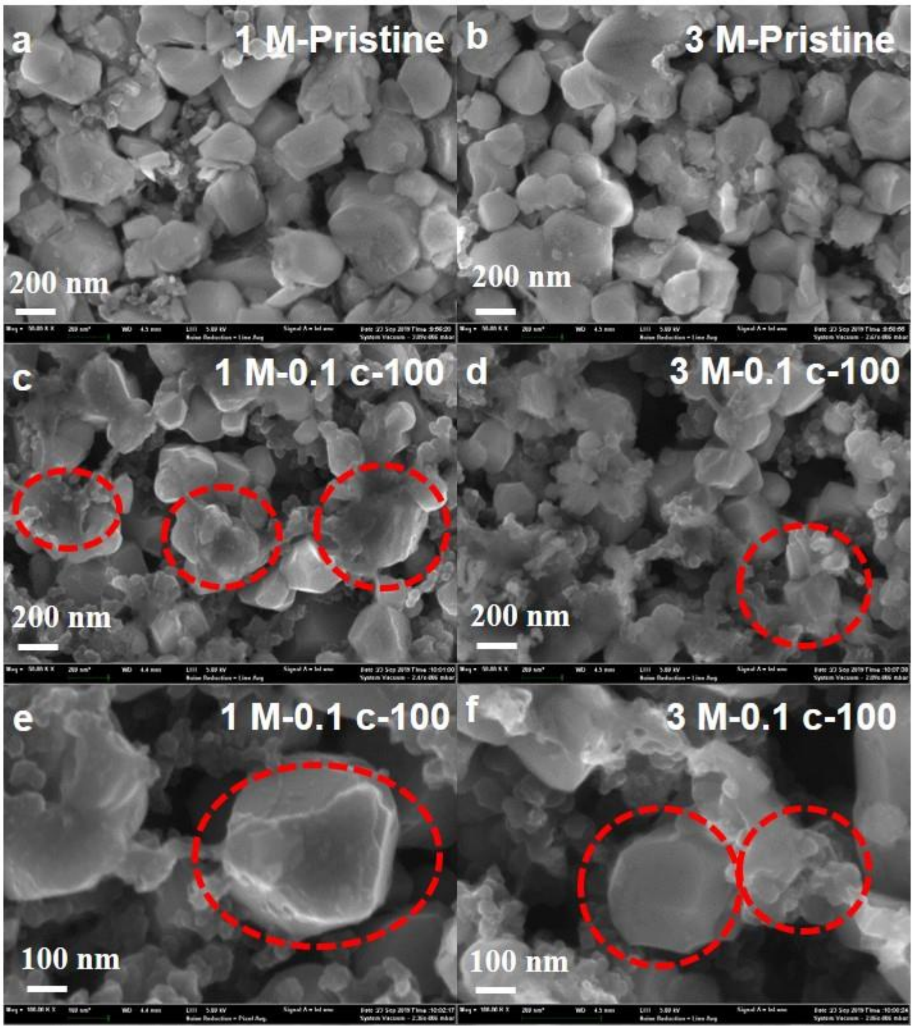

Supplementary Fig.10. The SEM images of electrode. The SEM images of the pristine electrode The SEM images of the at 0.1C in 1 M (a) and 3 M electrolytes (b); the SEM images of the 100 cycled electrode at 0.1 C in 1 M (c,e) and 3 M (d,f) electrolyte.

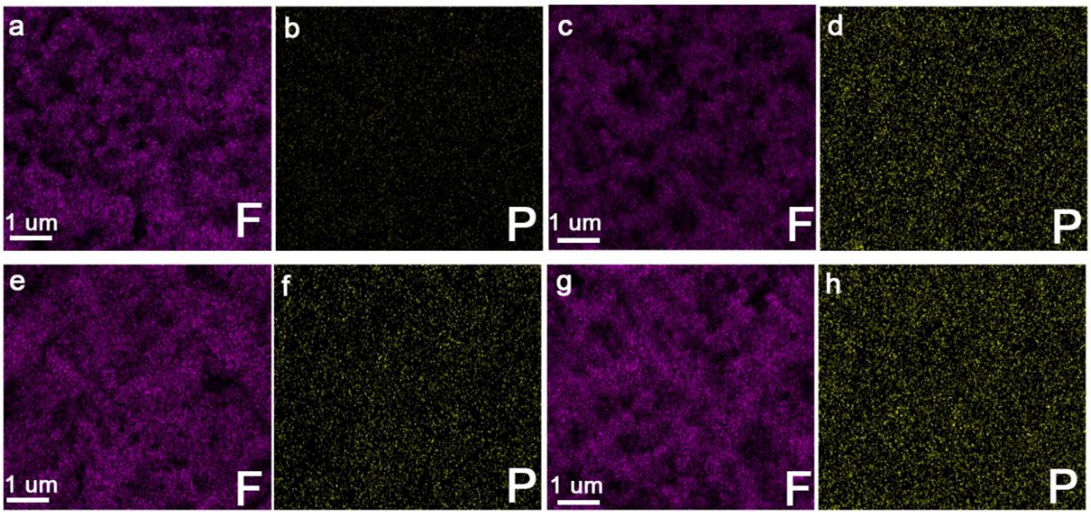

Supplementary Fig.11. EDS mapping of F、P for the pristine and 100 cycled at 0.1C .(a, b) The pristine of LLNFMO Shelved for 24 hours in 1 Melectrolyte; (c, d) LLNFMO after 100 cycles in 1 M electrolyte at 0.1C; (e, f) the pristine of LLNFMO shelved for 24 hours in 3 M electrolyte; (g, h）LLNFMO after 100 cycles in 3 M electrolyte at 0.1C .

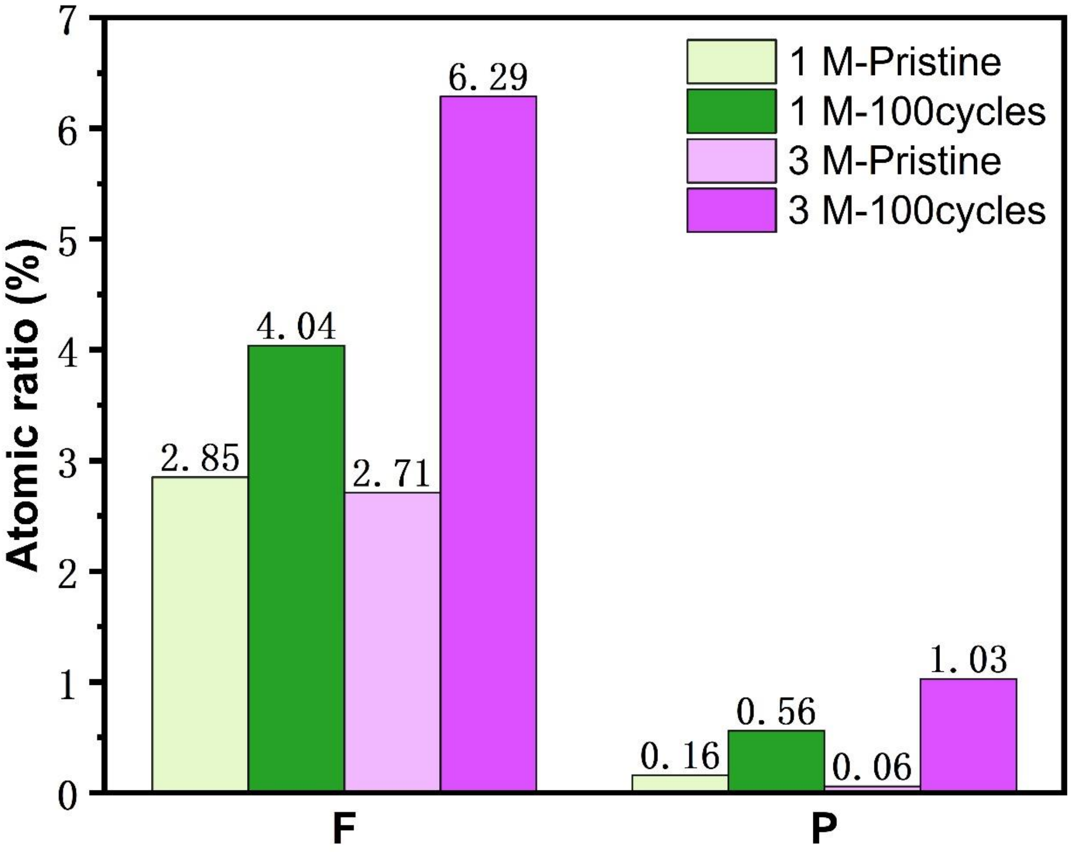

Supplementary Fig. 12. The atomic ratio of F and P on electrode surface. The atomic ratio of F and P on the surface of pristine (shelved for 24 hours in coin cell) and 100 cycled at 0.1C in 1 M and 3 M electrolytes.

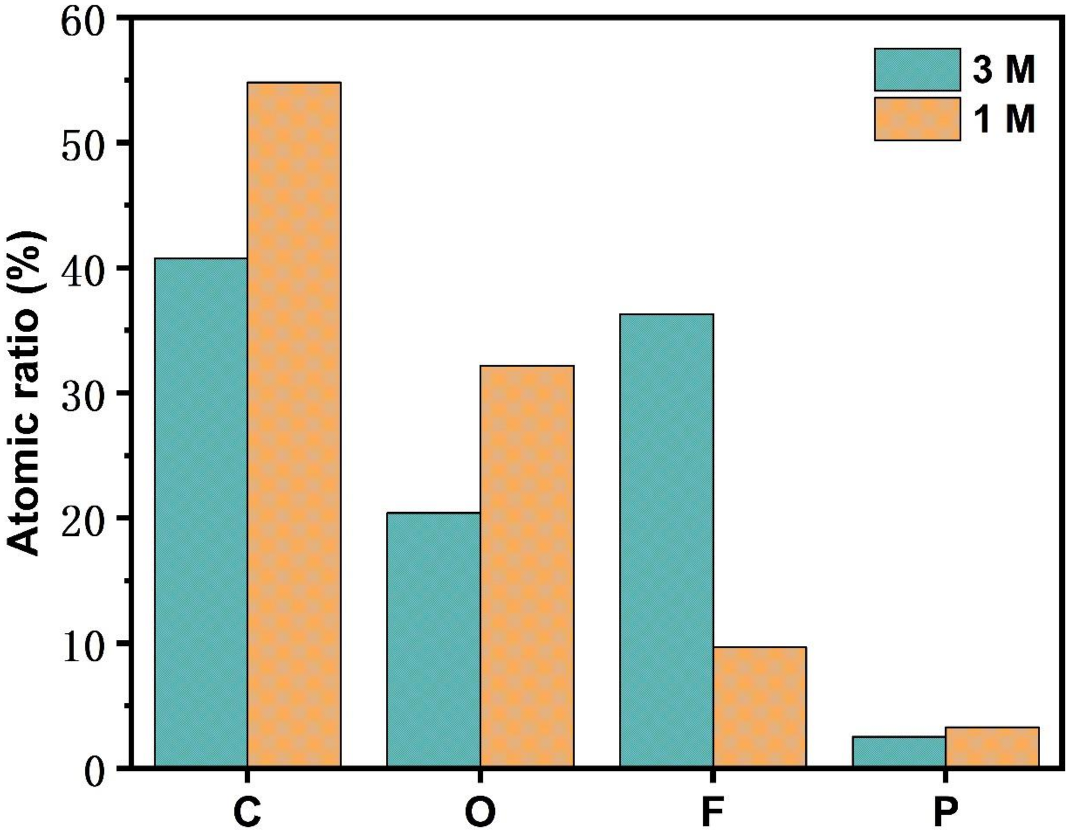

Supplementary Fig. 13. The element atomic ratio of cycled electrode. The atomic ratio of C, O, F, and P on the surface of electrode cycled in 1 M and 3 M electrolyte at 0.1C for 100 cycles obtained by XPS spectra.

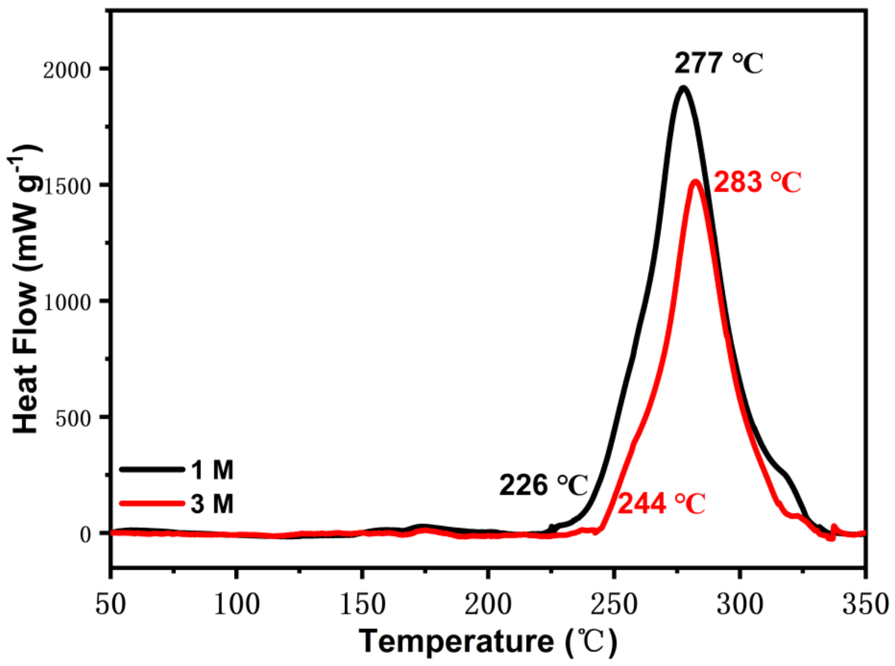

Supplementary Fig. 14. **DSC profile of electrode.** The DSC profile of LNFMO electrode after the first charging to 4.8V in 1 M and 3 M electrolyte.

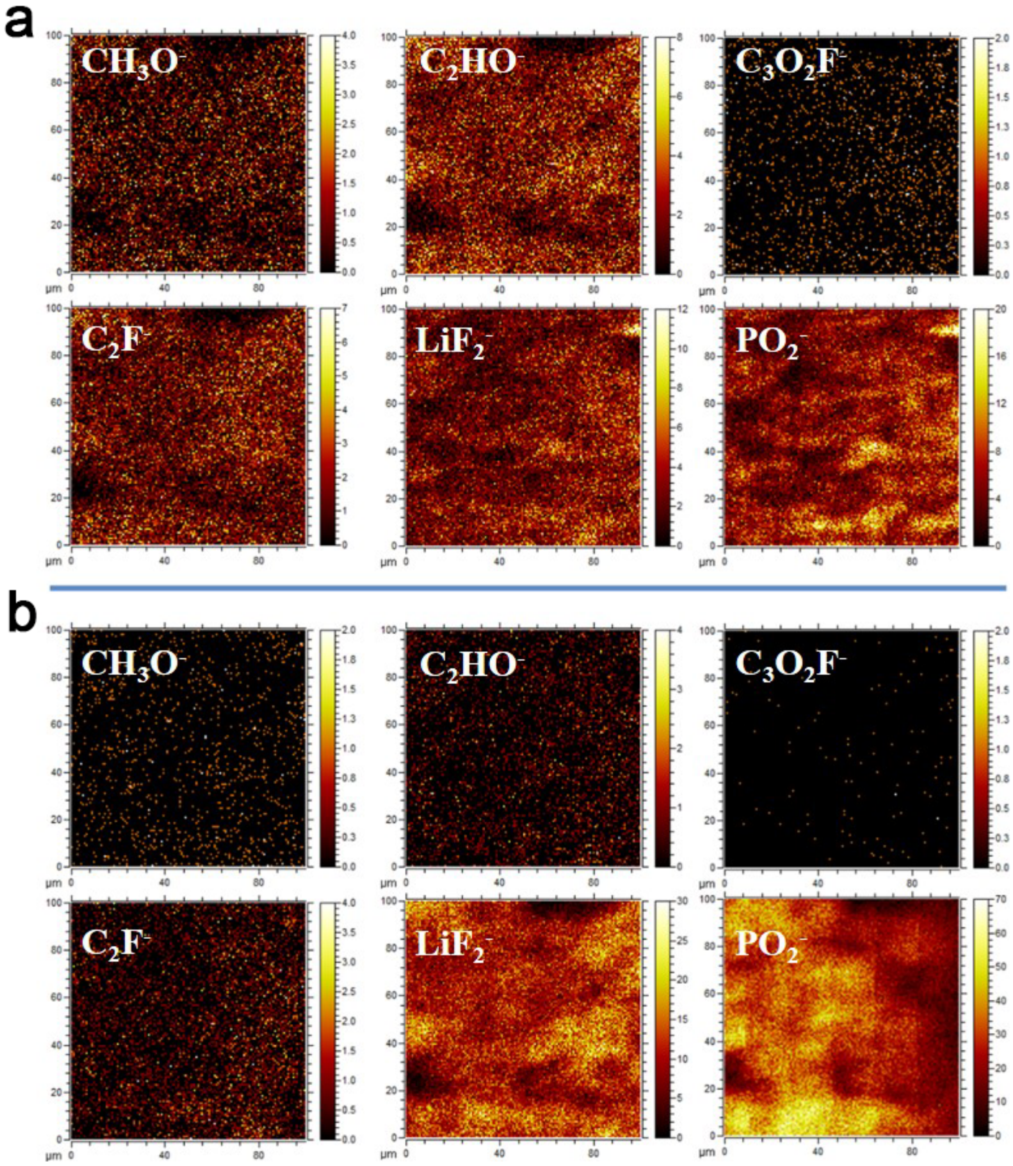

Supplementary Fig. 15. TOF-SIMS chemical maps of several typical second ion fragments. TOF-SIMS chemical maps of several typical second ion fragments with 25s of sputtering on electrodes cycled in 1 M electrolyte (a) and 3 M electrolyte (b), respectively.

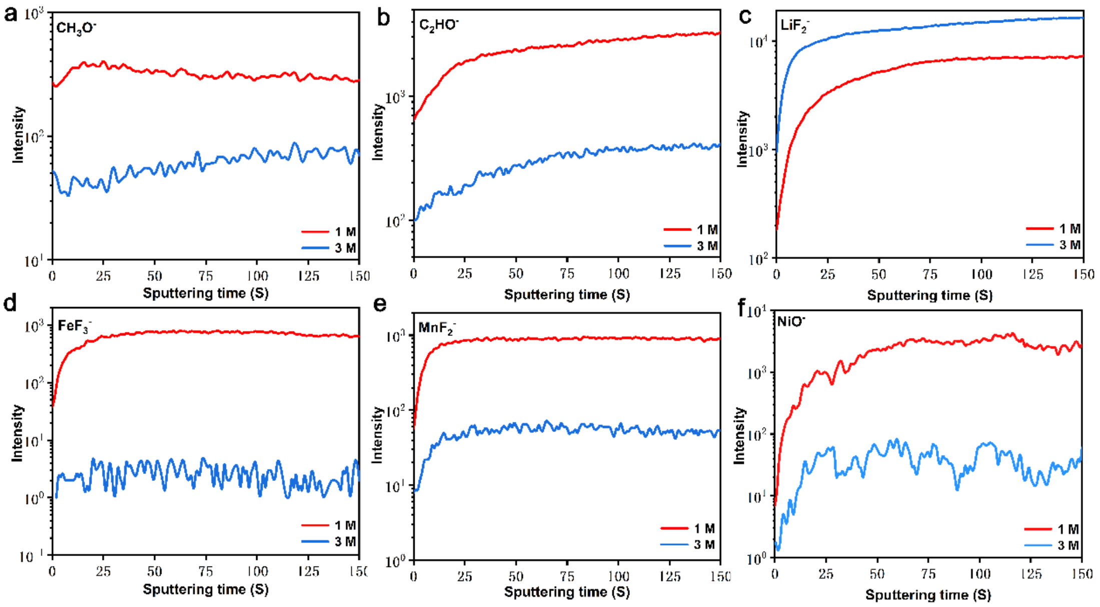

Supplementary Fig. 16. Corresponding depth profiling of second ion fragments on electrodes. The corresponding depth profiling of CH_3O^- (a) , C_2HO^- (b), LiF_2^- (c), FeF_3^- (d), MnF_2^- (e), and NiO^- (f) second ion fragments with 150s of sputtering on electrodes cycled in 1 M and 3 M electrolyte, respectively.

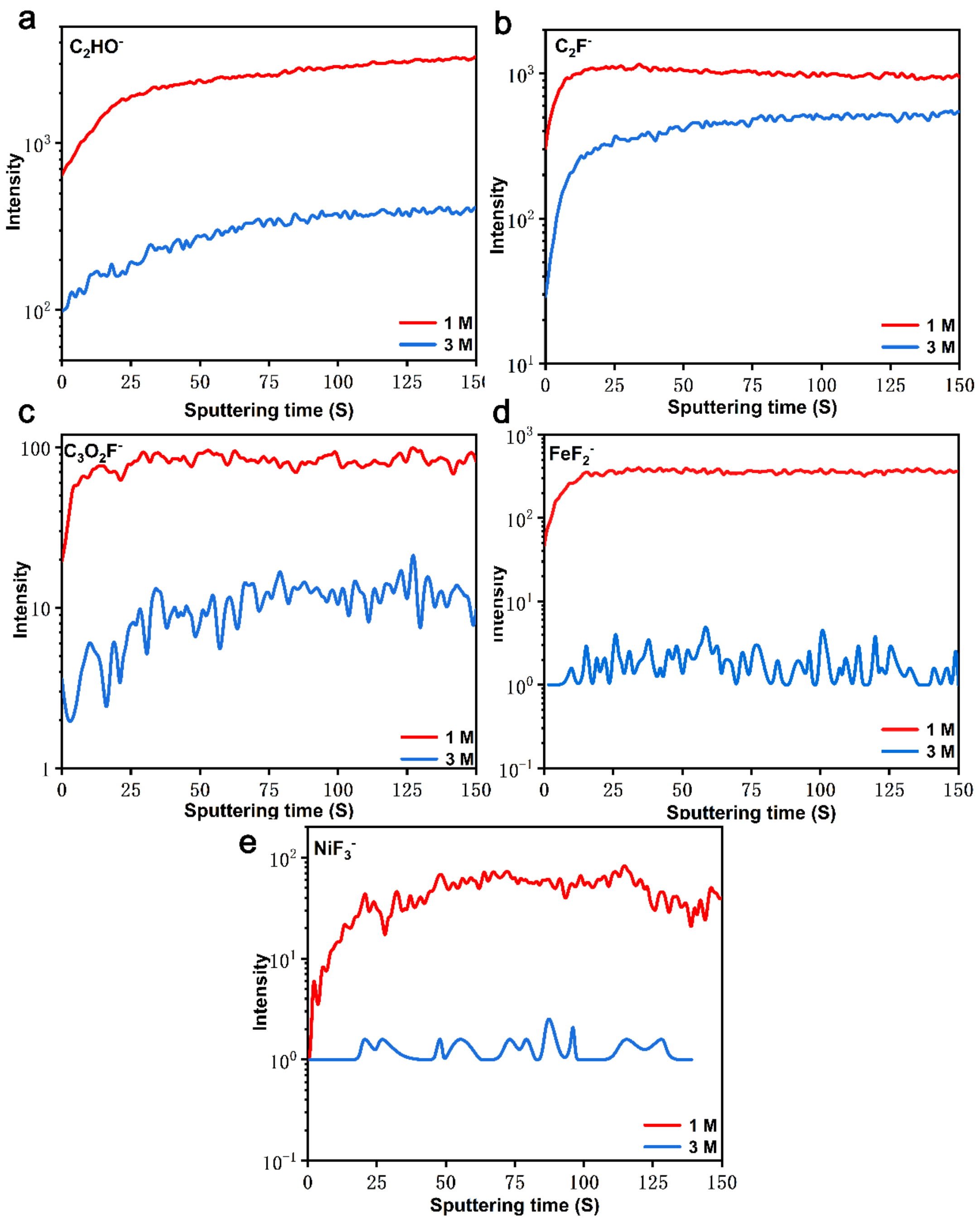

Supplementary Fig. 17. Depth profiling of second ion fragments of interest on electrodes. The depth profiling of C_2HO^- (a) , C_2F^- (b), $C_3O_2F^-$ (c), FeF_2^- (d), and NiF_3^- (e) second ion fragments with 150s of sputtering on electrodes cycled in 1 M and 3 M electrolyte, respectively.
