# Supplementary material for: Inhibition of transition metals dissolution in cobalt-free cathode with ultrathin robust interphase in concentrated electrolyte
Source: Nat Commun. 2020 Jul 20;11:3629. doi: 10.1038/s41467-020-17396-x (PMC7371675; doi:10.1038/s41467-020-17396-x)
Supplement: Supplementary file 2 — Peer Review File [file 41467_2020_17396_MOESM2_ESM.docx]

Peer Review Information

**Journal:** Nature Communications

**Manuscript title:** Inhibition of transition metals dissolution in cobalt-free cathode with ultrathin robust interphase in concentrated electrolyte

**Corresponding author name(s):** Prof Xinping Qiu

**Editorial notes:**

**none**

**Reviewer comments & decisions:**

**Reviewer comments, first version:**

Reviewer #1 (Remarks to the Author):

Recommendation: Publish elsewhere.

The manuscripts described the using of concentrated electrolyte to inhibit the dissolution of transition metals and to improve the cycling stability, CE, rate performance, and safety of the LNFMO. The experiments greatly support the role of concentrated electrolyte in forming the uniform and robust inorganic-rich CEI and improving the electrochemical performance. And the authors found that the dissolution of Fe rather than traditional acknowledged Mn is the main responsible for the low Coulombic efficiency of the LNFMO. It is a good piece of work. However, for one thing, there are many other reasons contributing to the low CE and poor cycling stability and the authors didn’t show direct evidence to confirm the dominant role of transition metal dissolution in reducing CE; For another, the authors also didn’t clarify the underlying reason of the less dissolution of Mn and the more serious dissolution of Fe in the LNFMO.

Moreover, the authors claim that this work provides a new idea forming a uniform and robust inorganic–rich CEI to effectively inhibit the dissolution of TM in a highly concentrated electrolyte. While, in the past years, it is widely reported that a high concentrated electrolyte will benefit the formation of a uniform and robust inorganic-rich CEI. And there are also lots of papers showing that using high concentrated electrolyte as well as localized high concentrated electrolyte is an effective approach in inhibiting the dissolution of transition metal ion during cycling for cathode materials of lithium/lithium-ion batteries. More importantly, the authors have not provided significant insight into how these electrolyte formulations function or how one would go about designing superior formulations to inhibit transition metal dissolution. Therefore, it is hardly to conclude that the paper provides the sort of significant conceptual advance in scientific understanding which makes it suit for publication in Nature communications.


Reviewer #2 (Remarks to the Author):

This work uses a combination of Lithium rich cathode material and concentrated EC based electrolyte and studies effect of salt concentration on various parameters of the battery. The topic of the communication is important for the scientific community working on next generation battery technologies given the sporadic studies dealing with both the aspect of Cathode Electrolyte Interphase (CEI) as well as concentrated electrolytes. The study prima-facie looks extensive. However,
there are certain questions which look either unanswered or incoherently conveyed. A major revision of the manuscript is required for it to be suitable for publication in Nature Communications. We would like authors to address the following points/queries.

1) In lines 46-48 it is mentioned "The anion-derived SEI exhibit not only higher stability but lower interfacial resistance compared with the solvent-derived SEI, which is beneficial to the electrochemical performance." Please provide evidence or a reference explaining the said phenomena

2) Concentrated electrolytes are known for offering reduced ionic conductivity. Please explain how the battery made of concentrated electrolyte in this study offers power performance at higher C-rates.

3) In lines 38-39, it is mentioned that voltage fade is due to the formation of disordered Ni-O rock salt structure. However, authors claim in line 181 that the voltage fade is reduced significantly in the case of concentrated electrolyte. It is not clear in what way salt concentration affects the voltage fade.

4) In lines 83-85 it is mentioned that "We clearly elucidate that the dissolution Fe ions from LNFMO is the dominant reason for the low CE rather than manganese during cycling in the conventional dilute electrolytes" In lines 184-185 highlight that "dissolution of TM is the main responsibility of poor
cycle stability and low CE of cathode materials"

However, in Fig 3d and Fig 4c it is shown that the Fe and Mn get deposited in almost proportional manner at different salt concentrations and for various cycle numbers. It does not show significant bias towards Fe or Mn to highlight that dissolution of Fe is the main reason. Please provide evidence supporting the argument made in lines 83-85.

5) In lines 134-136, it is mentioned that "The viscosity increases exponentially with the increase of salt concentration from 1 M to 5 M, while the corresponding ionic conductivity decreases sharply." Please fit the exponential functions and display appropriate fits.

6) In line 152, it is mentioned that "a unique solution structure like a 3D network is obtained in concentrated electrolyte." Please provide further details, and show supporting evidence in supporting info file.

7) In line 168, please bring clarity... is it end of cycle or "end of cycle life of battery"?

8) It seems the figure referred to in lines 172-174 and explanation provided mismatch.

9) Line 180, please show zoom of fig 3b in supporting info file to support your argument of "... noticeably enhanced"

10) Lines 200-201, please provide supporting evidence for Fe dissolution rather that of Mn dissolution.

11) Lines 214-216, regarding supplementary Figure 3. Please check cycles 20 to 25, the trend seems to be not normal. Please explain.

12) In line 217, it is mentioned that "This can be attributed to a novel Li+-conduction mechanism in concentrated
electrolyte" Please explain the mechanism and how it helps? Above what concentration, do you expect it to change?

13) Though it is not difficult to understand the content of the manuscript, it has a good scope for improvement. Please get it reviewed by native English speaker. Please try to avoid adjectives such as terrible, glorious, powerfully etc.

14) In line 220, it is mentioned that capacity drops to 150 mAh/g after 500 cycles, which is not good for EVs (see line 29).
Please discuss in what ways it can be improved to make Li rich electrodes a candidate for next generation batteries.

15) Line 227 - Please highlight/discuss c-rate dependent nature of dissolution

16) Line 234- Please explain supplementary Fig 5. It is not clear for what purpose 0.1 C and 1C are taken?

17) Line 256- Please elaborate and give details of baseline

18) Line 262- Please explain the reason for significant drop in specific discharge capacity during first 10 cycles as shown in Figure 4a.

19) Line 337- Please explain in what way presence of inorganic species is helping robustness of CEI.

20) Line 377- It does not look like a complete statement. Also, there are grammatical errors in the sentence following that. Please correct them.

21) Even though the cycle stability and safety can be improved by the formation of a uniform and better interphase at the electrode (suggested by several literature before), it would be of use to community to know how the salt concentration affects the formation of the CEI layer, Coulombic efficiency, and rate performance in detail. And also throw some light on effect of cycling on composition of electrolyte in the electrolyte phase.


Reviewer #3 (Remarks to the Author):

This is a well written paper with sufficient evidence to support their claims. In general, the high concentration electrolyte brings many benefits to the cell performance in terms of Coulombic efficiency, suppression of TM dissolution and deposition on the anode side, elevation of the high voltage stability et al. It has been a very hot topic in the past few years varying from using different solvents and different lithium salts and tested in various lithium-ion chemistries.

After I read through the manuscript, I feel the results reported in this paper is somehow new and important. The author used a Fe-substituted NMC material and reveled for the first time that Fe-dissolution is the main cause of the performance degradation of the cell. The prevention of Fe dissolution is due to the use of a high concentration LiPF6 based EC-EMC electrolyte.

However, I do have a few questions. (1) The CEI theory needs to be further clarified, meaning the mechanism of the formation. Why the high salt concentration helps the robust CEI formation?
(2) Figure 3 e and f the scale bar is different for the 1M and 3M cycled cathode. Although 1M cathode looks loose and less dense, its scale is very small. Fare comparison should be used.
(3) Half cell still used in this research. I'd like to see if this theory applies to the graphite full cell. (4) Figure 4, after long term cycling, the Fe-dissolution seems very similar for the 3M cycled cathode and 1 M one. How to explain this data?

Over all, I think this paper can be publication in Nat. Commun. after addressing all the above questions/comments.

**Author rebuttal, first version:**

Reviewer#1:

***The manuscripts described the using of concentrated electrolyte to inhibit the dissolution of transition metals and to improve the cycling stability, CE, rate performance, and safety of the LNFMO. The experiments greatly support the role of concentrated electrolyte in forming the uniform and robust inorganic-rich CEI and improving the electrochemical performance. And the authors found that the dissolution of Fe rather than traditional acknowledged Mn is the main responsible for the low Coulombic efficiency of the LNFMO. It is a good piece of work. However, for one thing, there are many other reasons contributing to the low CE and poor cycling stability and the authors didn’t show direct evidence to confirm the dominant role of transition metal dissolution in reducing CE; For another, the authors also didn’t clarify the underlying reason of the less dissolution of Mn and the more serious dissolution of Fe in the LNFMO.***

***Moreover, the authors claim that this work provides a new idea forming a uniform and robust inorganic–rich CEI to effectively inhibit the dissolution of TM in a highly concentrated electrolyte. While, in the past years, it is widely reported that a high concentrated electrolyte will benefit the formation of a uniform and robust inorganic-rich CEI. And there are also lots of papers showing that using high concentrated electrolyte as well as localized high concentrated electrolyte is an effective approach in inhibiting the dissolution of transition metal ion during cycling for cathode materials of lithium/lithium-ion batteries. More importantly, the authors have not provided significant insight into how these electrolyte formulations function or how one would go about designing superior formulations to inhibit transition metal dissolution. Therefore, it is hardly to conclude that the paper provides the sort of significant conceptual advance in scientific understanding which makes it suit for publication in Nature communications.***

Thank you very much for your valuable and constructive comments on our manuscript. We make the following explanations based on your comments, and we are eager for you to reconsider this work published in nature communications.

Cobalt-free lithium-rich cathode becomes a very competitive candidate for the next generation of cathode material. In order to get rid of cobalt, the Department of Energy in US awarded projects to develop the cobalt-free cathode materials for next-generation lithium-ion batteries in 2018. Fe-substituted Li-rich material has attracted a lot of attention due to the less voltage fade and the better capacity retention after the substitution of Co with Fe. However, it is puzzling that Fe-substituted lithium-rich materials exhibit lower Coulombic efficiency compared to Co-substituted lithium-rich materials. Many studies indicate that the dissolution of manganese in Mn-based cathode materials seriously affects the CE and cycle stability. The dissolution of the transition metal element causes the destruction and continuous growth of the interfacial film, and the consumption of active lithium ions leads to a reduction in CE. Iron-substituted cobalt-free lithium-rich materials have been widely reported, but none of them mention the CE. Later, we found that this material showed a lower CE. It is very urgent to explore the reason for the low CE. We found that the solubility of iron in Fe-substituted lithium-rich materials was much higher than that of manganese, which is significantly different from the phenomenon that the solubility of manganese is the highest (Fig. 3d, Fig. 4c). The dissolution of iron not only destroys the structure of the material but also causes the interface film to be continuously destroyed and grown, reducing the CE and cycle stability.

In recent years, highly concentrated electrolytes and localized highly concentrated electrolytes have been proven to effectively improve battery performance. However, these large amounts of these researches are mainly focused on the formation mechanism of SEI on anode side, and there are few studies on the composition change and distribution of CEI on cathode side. In this work, we conduct in-depth research and comparison of the changes in the composition and distribution of CEI in different electrolytes. We deeply explained the composition and structure of CEI in a highly concentrated electrolyte. We propose a mechanism for improving the performance of the battery with concentrated electrolyte from the effect on the composition and distribution of CEI components.

Reviewer#2:

Thank you very much for your valuable and constructive comments on our manuscript. The revisions you mentioned have been completed as followed.

***1) In lines 46-48 it is mentioned "The anion-derived SE I exhibit not only higher stability but lower interfacial resistance compared with the solvent-derived SEI, which is beneficial to the electrochemical performance." Please provide evidence or a reference explaining the said phenomena***

Reply: The corresponding new reference was added according to your advice highlighted in red in lines 66.
***2) Concentrated electrolytes are known for offering reduced ionic conductivity. Please explain how the battery made of concentrated electrolyte in this study offers power performance at higher C-rates.***
Reply: We all know that viscosity increases and ionic conductivity decrease with increasing salt concentration. We believe that there are two main reasons why concentrated electrolytes can provide higher rate performance at high rates. On the one hand, ion transport is directly linked to the rate performance of batteries. Traditional ion transport mechanisms (vehicle-type) achieve better ion transport performance by maximizing carrier density and mobility of dissociated free ions with a solvated sheath in dilute electrolytes ( as shown in Fig. 7a). However, this mechanism cannot be applied to concentrated electrolytes because the salt species exist more dominantly as associated states (for example, CIPs and AGGs). So far, there are few reports of related mechanisms. A hopping-type mechanism that Li^+^ moves from one anion to another through Lewis basic sites has been speculated, but this mechanism has not been demonstrated. On the other hand, the resistance of the Li^+^ passing through CEI is also directly linked to the rate performance of LNFMO cathode material. The unusual CEI formed in concentrated electrolyte is more critical than the ionic conductivity. This CEI formed in concentrated electrolyte is more stable and has lower impedance than formed in dilute electrolyte. Based on the above two aspects, we think the concentrated electrolytes can provide higher rate performance at high rates.
***3) In lines 38-39, it is mentioned that voltage fade is due to the formation of disordered Ni-O rock salt structure. However, authors claim in line 181 that the voltage fade is reduced significantly in the case of concentrated electrolyte. It is not clear in what way salt concentration affects the voltage fade.***
Reply：As mentioned in my manuscript, the formation of spinel-like and disordered NiO rock-salt structure are regarded as the origin of voltage fade. On the one hand, we found that the substitution of Co with Fe in Li-rich (Fe-Li-rich) materials can effectively suppress voltage fade by inhibiting the formation of peroxy bonds and phase transformation in our previous works. In this work, the phenomenon of effectively suppressing voltage decay also occurred on Li_1.2_Ni_0.15_Fe_0.10_Mn_0.55_O_2_, especially cycled in high salt concentration electrolyte (Fig. 3b). To investigate the mechanism of less voltage fade, we carried out various post-mortem analysis. (e.g. Raman、TOF-SIMS) We surprisingly found that the formation of spinel-like and disordered NiO rock-salt structure were greatly suppressed in 3 M electrolyte (Fig. 6, Supplementary Fig. 7**).** Our further investigation found the robust CEI formed in concentrated electrolyte effectively inhibited metal dissolution and protected the stability of the material structure, thereby reducing phase transitions. As a result, the voltage fade is reduced in the concentrated electrolyte.
***4) In lines 83-85 it is mentioned that "We clearly elucidate that the dissolution Fe ions from LNFMO is the dominant reason for the low CE rather than manganese during cycling in the conventional dilute electrolytes" In lines 184-185 highlight that "dissolution of TM is the main responsibility of poor cycle stability and low CE of cathode materials"
However, in Fig 3d and Fig 4c it is shown that the Fe and Mn get deposited in almost proportional manner at different salt concentrations and for various cycle numbers. It does not show significant bias towards Fe or Mn to highlight that dissolution of Fe is the main reason. Please provide evidence supporting the argument made in lines 83-85.***
Reply: Firstly, thank you for your valuable and thoughtful comment. As I wrote in my introduction, the Mn^4+^ ions in Li-rich Mn-based materials tend to be reduced to Mn^3+^ ions due to the Jahn-Teller distortion. The Mn^3+^ on the surface is extremely unstable and easily tends to generate more soluble Mn^2+^ by disproportionate decomposition. Considering the highest proportion of Mn in TM and the characteristics of easy dissolution of Mn^2+^, the dissolution and deposition of Mn in Li-rich Mn-based materials is the most serious far beyond other transition metals (e.g. Ni，Co, Al, etc.). However, we found that the dissolution and deposition of Fe far exceeds that of Mn. In response to this anomaly, we conducted a variety of test analyses, and all the results showed that the dissolution of iron was the most severe. Super-oxidative Fe^4+^ ions are generated on the particle surface when charged to 4.8 V and then react with the electrolyte to dissolve in the electrolyte. We induce that this causes the Fe to dissolve far more than Mn. As we can see in the results, the dissolution and deposition of Fe is the most serious after a long cycle. After considering the relationship between the dissolution of transition metals and the electrochemical performance of LNFMO, we elucidate that the dissolution Fe ions from LNFMO is the dominant reason for the low CE rather than manganese during cycling in the conventional dilute electrolyte.

***5) In lines 134-136, it is mentioned that "The viscosity increases exponentially with the increase of salt concentration from 1 M to 5 M, while the corresponding ionic conductivity decreases sharply." Please fit the exponential functions and display appropriate fits.***
Reply: Thank you very much for pointing out the improper description of functional equation. Viscosity does not increase exponentially with salt concentration, but may follow the Jones-Dole equation.

$$\text{y=1+a}x^{\text{1/2}}+bx+cx^{2}+dx^{3}$$

The result of the fitting is shown in the following equation.

$$\text{y}=1+7.88x^{1/2}-9.06x+2x^{2}+0.62x^{3}$$

We have revised this error and the changes were highlighted in red in our revised manuscript.
***6) In line 152, it is mentioned that "a unique solution structure like a 3D network is obtained in concentrated electrolyte." Please provide further details, and show supporting evidence in supporting info file.***
Reply: We apologize for our imprecise description. The structure of electrolytes with high salt concentration is significantly different from that of ordinary dilute electrolytes. Such similar results have been reported a lot before, so we are directly citing others’ works here. We conclude that the unique structure of the concentrated electrolyte is based on our Raman results and cites the work of others. We have revised our imprecise description in our revised manuscript.
***7) In line 168, please bring clarity... is it end of cycle or "end of cycle life of battery"?***
Reply: We have to apologize for giving you so much trouble for reviewing our manuscript. It is end of cycle, not the end of life of battery.
***8) It seems the figure referred to in lines 172-174 and explanation provided mismatch.***
Reply: We apologize for giving you trouble for reviewing our manuscript. Fig. 3c shows the initial charge-discharge curve at 0.1C in 1 M and 3 M electrolyte. The CE is presented in Fig.3a. We have revised these mistakes thoroughly and the changes were highlighted in red in our revised manuscript.
***9) Line 180, please show zoom of fig 3b in supporting info file to support your argument of "... noticeably enhanced"***
Reply: The corresponding zoom of Fig.3b was presented in Supplementary Fig. 2 according to your advice. We have revised these mistakes thoroughly and the changes were highlighted in red in our revised manuscript.
***10) Lines 200-201, please provide supporting evidence for Fe dissolution rather that of Mn dissolution.***
Reply: We apologize for giving you trouble for reviewing our manuscript. This question is the same as the fourth one, already answered in the fourth one.
***11) Lines 214-216, regarding supplementary Figure 3. Please check cycles 20 to 25, the trend seems to be not normal. Please explain.***
Reply: We found that the rate performance was significantly improved after using concentrated electrolytes. However, we also found the discharge capacity of LNFMO cycled at 5 C in 5 M electrolytes reduced compared to 3 M and 4 M electrolytes. The rate performance is significantly improved due to the novel Li^+^-conduction mechanism. The viscosity of the 5 M electrolyte is far larger than 3 M and 4 M electrolytes, which increases the charge transfer resistance. As a result, we see that the rate performance cycled at 2 C in 5 M electrolytes is poor compared to other electrolytes.
***12) In line 217, it is mentioned that "This can be attributed to a novel Li+-conduction mechanism in concentrated electrolyte" Please explain the mechanism and how it helps? Above what concentration, do you expect it to change?***
Reply: The rate performance of a battery is intuitively associated with the ionic conductivity of the electrolyte and CEI, although it is actually determined by the resistance of not only Li^+^ transport in the electrolyte but also Li^+^ passing through the electrolyte/electrode interface. In this article, the increased electrolyte viscosity hinders lithium ion transport. However, here are some novel findings in concentrated electrolyte. Traditional ion transport mechanisms (vehicle-type) achieve better ion transport performance by maximizing carrier density and mobility of dissociated free ions with a solvated sheath in dilute electrolytes. (Fig. 7a) However, this mechanism cannot be applied to concentrated electrolytes because the salt species exist more dominantly as associated states (for example, CIPs and AGGs). So far, there have been few reports of related mechanisms and they are not very satisfactory. A hopping-type mechanism that Li^+^ moves from one anion to another through Lewis basic sites has been speculated, but this mechanism has not been demonstrated.

We apologize for the imperfect expression of this sentence. The resistance of CEI is also critical to the rate performance. We have explained in the second question. We have revised these mistakes thoroughly and the changes were highlighted in red in our revised manuscript.

We believe that sufficient associated states (e.g. AGGs) can be formed in the electrolyte to help improve rate performance.

***13) Though it is not difficult to understand the content of the manuscript, it has a good scope for improvement. Please get it reviewed by native English speaker. Please try to avoid adjectives such as terrible, glorious, powerfully etc.***
Reply: We apologize for giving you trouble for reviewing our manuscript. We have made thorough revises to avoid these problems.

***14) In line 220, it is mentioned that capacity drops to 150 mAh/g after 500 cycles, which is not good for EVs (see line 29).
Please discuss in what ways it can be improved to make Li rich electrodes a candidate for next generation batteries.***
Reply: We really appreciate your comment. We also noticed this problem in the experiment. In this article, we find the cause of low Coulombic efficiency and improve electrochemical performance by building a robust CEI with concentrated electrolyte. The LNFMO exhibits a higher reversible specific capacity and better cyclic stability in concentrated electrolyte than dilute electrolyte. The LNFMO exhibits 150mAh/g at 2 C rate after 500 cycles, seems not good for EVs. There is no denying that there is still a certain distance from commercial. However, there is still much room for improvement rate performance and cyclic stability. This is exactly what we will do next. At present, we have got some new results in this area, and we have been working hard to make the performance of LNFMO meet the commercial needs.

***15) Line 227 - Please highlight/discuss c-rate dependent nature of dissolution***
Reply: We really appreciate your comment. We have discuss the C-rate dependence nature of dissolution and the changes were highlighted in red in the revised manuscript. ***16) Line 234- Please explain supplementary Fig 5. It is not clear for what purpose 0.1 C and 1C are taken?***
Reply: The coin cells were firstly cycled at 0.1C for 3 cycles to activate the Li_2_MnO_3_ and form a passivation film on the surface of electrodes before being cycled at higher C-rate.
***17) Line 256- Please elaborate and give details of baseline***
Reply: We used SEM-EDS to measure the element content on the surface of the electrodes, one is pristine, and the other is after 100 cycles. The pristine electrodes were shelved for 24 hours in 1 M and 3 M electrolytes, respectively. We choose to record the atomic ratio of these elements, including carbon, oxygen, fluorine, phosphorus, manganese, iron, and nickel. The supplementary Fig.10. shows the SEM-EDS mapping of F、P for the pristine and 100 cycled at 0.1C. The supplementary Fig. 11. Shows the atomic ratio of F and P on the surface of pristine (shelved for 24 hours in coin cell) and 100 cycled at 0.1C in 1 M and 3 M electrolytes.
***18) Line 262- Please explain the reason for significant drop in specific discharge capacity during first 10 cycles as shown in Figure 4a.***
Reply: In this article, the coin cells were firstly cycled at 0.1C for 3 cycles to activate the Li_2_MnO_3_ and form a passivation film on the surface of electrodes before being cycled at higher C-rate. The polarization increases significantly including ohmic polarization and electrochemical polarization when charged and discharged at a large current, as a result, the capacity drops suddenly. At the same time, rapid extraction- insertion of Li^+^ destroys the previous passivation film, and a new passivation film needs to be formed again. Lithium ions in the center of the particle are difficult to diffuse into the particle surface, and the surface of the particle is more prone to overcharging, which causes oxygen evolution and the lattice structure to collapse, resulting in a decrease in capacity.
***19) Line 337- Please explain in what way presence of inorganic species is helping robustness of CEI.***
Reply: The HRTEM images of CEI indicate that the CEI formed in concentrated electrolyte is ultra-thin and flat. The XPS analyses indicate that the surface composition of the CEI film is almost all LiF. Furthermore, the intensity of LiF_2_^-^ on the surface of electrode cycled in 3 M electrolyte is far higher than in 1 M electrolyte.

All these results of HRTEM, XPS and TOF-SIMS analyses indicate the main component of the inorganic species is LiF. The CEI layer is supposed to be a complicated multilayer structure. There are obvious differences between the carbonate-derived and LiF-rich CEI. The interface between the carbonate-derived CEI and dilute electrolyte is rugged, where the outer layer is mainly composed of organic species (e.g., RCOxLi) and some dissolved products. However, the interface between the LiF-rich CEI and concentrated electrolyte is flat and the outer layer is mainly composed of LiF.

We think it is the outermost layer of LiF that helps robustness of CEI.
***20) Line 377- It does not look like a complete statement. Also, there are grammatical errors in the sentence following that. Please correct them.***
Reply: We really appreciate your comment. We have revised this error and the changes were highlighted in red in our revised manuscript.
***21) Even though the cycle stability and safety can be improved by the formation of a uniform and better interphase at the electrode (suggested by several literature before), it would be of use to community to know how the salt concentration affects the formation of the CEI layer, Coulombic efficiency, and rate performance in detail. And also throw some light on effect of cycling on composition of electrolyte in the electrolyte phase.***

Reply: We greatly appreciate your review of our work. Your suggestions are very good, with your help we have a deeper understanding of this work. With your encouragement, we will do more in this area to solve some of the deficiencies in this work.

Reviewer #3

Thank you very much for your valuable and constructive comments on our manuscript. The revisions you mentioned have been completed as followed.

(1) ***The CEI theory needs to be further clarified, meaning the mechanism of the formation. Why the high salt concentration helps the robust CEI formation?***

Reply: We appreciate your suggestion. Your suggestion is very helpful for our next work. There is no doubt that the CEI formation mechanism in concentrated electrolyte must be further discussed in depth. At present, there are few studies on the mechanism of CEI formation in concentrated electrolytes. This is very important and meaningful work. We plan to focus on this work recently and cooperate with colleagues in the field of theoretical computing. I believe that this independent work will be published soon.

(2) ***Figure 3 e and f the scale bar is different for the 1M and 3M cycled cathode. Although 1M cathode looks loose and less dense, its scale is very small. Fare comparison should be used.***

Reply: We appreciate your comment. We have revised this error and the changes were highlighted in red in our revised manuscript.

***(3) Half cell still used in this research. I'd like to see if this theory applies to the graphite full cell.***

Reply: We regret that all the test cells in this article are half cells. Your suggestion, whether the theory applies to the graphite full cells is very good. We also want to know the result urgently. Unfortunately, it will not be possible to return to school to supplement this experiment for the next period due to the well-known epidemic effect. We plan to arrange this part of the work into the work in the first question and research this work in exploring the CEI formation mechanism.

***(4) Figure 4, after long term cycling, the Fe-dissolution seems very similar for the 3M cycled cathode and 1 M one. How to explain this data?***

Reply: We are very sorry that our data has troubled your review. The Fig.4 shows the amount of transition metal deposited on the different cycled lithium plates in 1 M and 3 M electrolytes at 2 C. We can see that the amount of transition metal deposition increases significantly as the number of cycles increases cycled in 1 M electrolyte. And the amount of Fe deposited in 1 M electrolyte is much larger than that in 3 M electrolyte. In contrast, the deposition of transition metals is tremendously suppressed in 3 M electrolyte. Although the deposition amount of the transition metals in the 3 M electrolyte also increases with the cycle, it is far less than that in the 1 M electrolyte. Therefore, we believe that the dissolution of transition metal is significantly suppressed with 3 M electrolyte.

**Reviewer comments, second version:**

Reviewer #1 (Remarks to the Author):

1. It is better to supply molecular dynamics simulation data to describe the diluent and concentrated electrolytes. This will help to explain the formation mechanism of CEIs in 1M and 3M electrolytes.
2. There are two “Supplementary Fig. 2” in Supplementary information. The figures described in manuscript don’t match with the ones presented in Supplementary information.
3. For the rate performance, authors claim that the higher reversible discharge capacities at all different C-rate in the cells with concentrated electrolytes are attributed to a novel Li+ conduction mechanism in concentrated electrolyte. Please give more explain about the novel Li+ conduction mechanism.
4. In Supplementary Fig. 3, the rate performance, why 5M electrolyte shows the best rate capability at rates of 0.1 C-1C with the highest viscosity and lowest conductivity? Why 4M electrolyte shows the highest capacity at 2C?
5. Compared Figure 3d and Figure 4c, it seems that the amount of TM deposited in the Li plates after 100 cycles at 2C is much lower than that at 0.1 C. While the authors also claim that the deintercalation of lithium ions at high rates will accelerate the dissolution of TMs. It’s confusing. Please provide some comments.
6. In Raman spectra, Supplementary Fig. 7, it would be better if some quantitative data can be given to support the description of “the less components of the spinel phase are observed in 3 M electrolyte”.
7. Normally, high temperature will accelerate the TM dissolution and lead to advanced failure. Compared the 2C rate cycling at 25 (Fig. 4a) and 40 °C (supplementary Fig. 8) with 1M electrolyte, the cell at 25°C shows a sharp drop at around 250th cycle and the capacities drop to 100mAh/g at around 300th cycle. While, for the cell at 40 °C, no obvious drop before 300 cycles and the capacities drop to 100mAh/g at around 420th cycle. Please give reasonable explain.
8. In Figure 5c,d, why the peak intensities of Fe 2p and Mn 2p of the 1st cycles are higher than those of the 100th cycles for the 3M electrolyte?
9. Please give some comments on the difference of the electrolyte decomposition mechanisms in 1M and 3M electrolytes which leads to the inorganic-rich CEI in 3M electrolyte but more organic CEI in 1M electrolyte, the completion among EC/EMC/DMC solvents, and the completion between solvents and salt.
10. In Figure 6a and 6b, if the colors are consistent (CH3O- and LiF2-), the readability would be better.
11. It is claimed that “the higher intensity of FeF2-, FeF3-, MnF2-, and NiF3- fragments on the electrode surface cycled in 1 M electrolyte are presented clearly”. But in Figure 6 and Supplementary Figures 14-16, no any information of NiF3-.


Reviewer #2 (Remarks to the Author):

Authors have implemented the changes as per the review comments to a satisfactory level. However, the manuscript needs to be thoroughly checked once by a native speaker for language corrections before publication.


Reviewer #3 (Remarks to the Author):

The authors have sufficiently addressed all my questions/comments in a good amount of details. The revised manuscript can be accepted for publication in Nat.Comm.

**Author rebuttal, second version:**

Reviewer#1:

Thank you very much for your valuable and constructive comments to improve the quality of this manuscript and our work. We have responded to your comments point-to-point.

1. **It is better to supply molecular dynamics simulation data to describe the diluent and concentrated electrolytes. This will help to explain the formation mechanism of CEIs in 1M and 3M electrolytes.**

Reply：We are very grateful for your suggestion. Your suggestion is very helpful for our next work. There is no doubt that the CEI formation mechanism in concentrated electrolyte must be further discussed in depth. At present, there are few studies on the mechanism of CEI formation in concentrated electrolytes. This is a very important and meaningful work. We plan to focus on this work recently and cooperate with colleagues in the field of theoretical computing. I believe that this independent work will be published soon.

2. **There are two “Supplementary Fig. 2” in Supplementary information. The figures described in manuscript don’t match with the ones presented in Supplementary information.**

Reply: We are very sorry for my mistake, we have completely and carefully changed this information.

3. **For the rate performance, authors claim that the higher reversible discharge capacities at all different C-rate in the cells with concentrated electrolytes are attributed to a novel Li+ conduction mechanism in concentrated electrolyte. Please give more explain about the novel Li+ conduction mechanism.**

Reply: We all know that viscosity increases and ionic conductivity decrease with increasing salt concentration. We believe that there are two main reasons why concentrated electrolytes can provide higher rate performance at high rates. On the one hand, ion transport is directly linked to the rate performance of batteries. Traditional ion transport mechanisms (vehicle-type) achieve better ion transport performance by maximizing carrier density and mobility of dissociated free ions with a solvated sheath in dilute electrolytes ( as shown in Fig. 7a). However, this mechanism cannot be applied to concentrated electrolytes because the salt species exist more dominantly as associated states (for example, CIPs and AGGs). So far, there are few reports of related mechanisms. A hopping-type mechanism that Li^+^ moves from one anion to another through Lewis basic sites has been speculated, but this mechanism has not been demonstrated. On the other hand, the resistance of the Li^+^ passing through CEI is also directly linked to the rate performance of LNFMO cathode material. The unusual CEI formed in concentrated electrolyte is more critical than the ionic conductivity. This CEI formed in concentrated electrolyte is more stable and has lower impedance than formed in dilute electrolyte. Based on the above two aspects, we think the concentrated electrolytes can provide higher rate performance at high rates.

**4. In Supplementary Fig. 3, the rate performance, why 5M electrolyte shows the best rate capability at rates of 0.1 C-1C with the highest viscosity and lowest conductivity? Why 4M electrolyte shows the highest capacity at 2C?**

Reply: We found that the rate performance was significantly improved after using concentrated electrolytes. However, we also found the discharge capacity of LNFMO cycled at 5 C in 5 M electrolytes reduced compared to 3 M and 4 M electrolytes. The rate performance is significantly improved due to the novel Li^+^-conduction mechanism. However, the viscosity of the 5 M electrolyte is far larger than 3 M and 4 M electrolytes, which significantly increases the charge transfer resistance. As a result, we found that the rate performance cycled at 2 C in 5 M electrolytes is poor compared to other electrolytes. As for the highest capacity shown in 4 M electrolyte at 2 C, it is the result of balanced novel Li^+^-conduction mechanism and charge transfer resistance.

**5. Compared Figure 3d and Figure 4c, it seems that the amount of TM deposited in the Li plates after 100 cycles at 2C is much lower than that at 0.1 C. While the authors also claim that the deintercalation of lithium ions at high rates will accelerate the dissolution of TMs. It’s confusing. Please provide some comments.**

Reply: Thank you very much for this comment. During high-rate cycling, rapid intercalation and deintercalation of lithium ions cause severe lattice expansion/contraction, accelerate material rupture and accelerate phase transition, which will accelerate the dissolution of transition metals. Besides, the reaction of the cathode material with the electrolyte will also cause the transition metal to dissolve. It takes 84 days to cycle 100 cycles at 0.1C, but 500 days at 2C for 21 days. The amount of transition metal dissolved is still very low when cycling at 0.1C for 25 cycles. We added this data and added it to **Supplementary Fig. 5.** The long-term reaction of cathode material with electrolyte accelerates the dissolution of transition metals. We believe that the long-term cycling at 0.1C leads to a greater amount of transition metal dissolved.

**Supplementary Fig. 5.** The amount of transition metals deposited on lithium metal counter electrode after 25 cycles at 0.1 C（a） and 500 cycles at 5 C (b), respectively.

**6. In Raman spectra, Supplementary Fig. 7, it would be better if some quantitative data can be given to support the description of “the less components of the spinel phase are observed in 3 M electrolyte”.**

Reply: Thanks for your kind suggestion. The main peak near 600 cm^-1^ (A1g peak )can be deconvoluted into two peaks, which are assigned into the layered LNFMO at 595 cm^-1^ and the spinel peak at 630 cm^-1^, respectively. We can intuitively find that the material after cycling in the 3M electrolyte has fewer spinel phase components by calculating the area ratio of the two peaks. According to your suggestions, we have revised in the supplementary information, and the results are as follows. We have made corresponding changes in the manuscript and marked with red.

**Supplementary Fig. 8.** The Raman spectra of pristine LLNFMO and the electrodes after 200 cycles at 1C in 1 M and 3 M electrolyte.

**7. Normally, high temperature will accelerate the TM dissolution and lead to advanced failure. Compared the 2C rate cycling at 25 (Fig. 4a) and 40 °C (Supplementary Fig. 8) with 1M electrolyte, the cell at 25°C shows a sharp drop at around 250th cycle and the capacities drop to 100mAh/g at around 300th cycle. While, for the cell at 40 °C, no obvious drop before 300 cycles and the capacities drop to 100mAh/g at around 420th cycle. Please give reasonable explain.**

Reply: Your comment is very good. We have tried charging and discharging at higher temperatures, such as 50 ℃, 55 ℃ and 60 ℃. However, after cycling for dozens of cycles, the battery capacity quickly decays. Later, when we adjusted the temperature to 40 ℃, we found that the battery could circulate stably. It also shows relatively better cycle performance compared to 25 ℃. Cui et al.^1^ demonstrated enhanced performance in lithium metal batteries operated at elevated temperatures. Average Coulombic efficiency of 99.3% is obtained and more than 300 stable cycles are realized in an ether-based electrolyte at 60 ℃. They showed that high-temperature battery operation can lead to a favorable SEI nanostructure, enlarge Li particle size and provide faster kinetics, resulting in enhanced CE and cycling stability. We speculate that the more stable CEI may generate on the positive electrode surface and the more stable SEI forms on the lithium metal surface, which suppresses the dissolution of the positive electrode material and the decomposition of the electrolyte. As a result, the cells exhibited better cycle performance at 40 ℃.

1. Wang, J. *et al.* Improving cyclability of Li metal batteries at elevated temperatures and its origin revealed by cryo-electron microscopy. *Nature Energy* **4**, 664-670 (2019).

**8. In Figure 5c,d, why the peak intensities of Fe 2p and Mn 2p of the 1st cycles are higher than those of the 100th cycles for the 3M electrolyte?**

Reply: The peak intensity of Fe 2p and Mn 2p of the 1st cycles are similar to that of the pristine material in 1 M and 3 M electrolytes. However, a robust and uniform CEI is formed on the electrode surface after 100 cycles in 3 M electrolyte, so that XPS is difficult to detect, but, the uneven and fragile organic-rich CEI layer is generated on the outmost surface of the electrode in 1 M electrolyte. Finally, the peak intensities of Fe 2p and Mn 2p of the 100th cycles in 1M electrolyte are higher than those of the 100th cycles in the 3M electrolyte.

**9. Please give some comments on the difference of the electrolyte decomposition mechanisms in 1M and 3M electrolytes which leads to the inorganic-rich CEI in 3M electrolyte but more organic CEI in 1M electrolyte, the completion among EC/EMC/DMC solvents, and the completion between solvents and salt.**

Reply: The CEI layers on positive electrodes generally consist surprisingly of similar solvent and salt decomposition products as those found on negative electrodes. The difference between the Fermi level of the electrode and the highest occupied molecular orbital (HOMO) or the lowest unoccupied molecular orbital (LUMO) levels of the electrolyte governs the thermodynamic stability of the electrolyte on the electrode and the driving force to form the CEI layer. A negative electrode with a Fermi level higher in energy than the LUMO of the electrolyte will have a driving force to reduce the electrolyte. On the positive side, there will be a driving force for electrolyte oxidation if the Fermi level of the electrode is lower in energy than the HOMO of the electrolyte. Pure carbonate solvents are used commonly in Li-ion batteries such as EC, EMC, and DMC, whose LUMO levels close to the Li+/Li couple. No significant difference is noted for the LUMO levels of pure EC and DMC, indicating that changing the carbon chain attached to the carbonate group does not affect the LUMO level. However, salt anions have been experimentally determined to play an important role in the stability of electrolytes on the negative electrode. The HOMO levels of pure carbonate solvents are close to 7 V Li/Li^+^. When different salt anion is added to the solvent, the HOMO levels of the electrolytes are increased by different magnitudes.

Usually, the LUMO is dominated by solvent molecules; hence, the solvent molecules are predominantly reduced and contribute to SEI formation. As the concentration increases, the solvation has become completely different, and the number of free solvent molecules is drastically decreased and the aggregates (AGGs) increases. The extensive coordination to Li^+^ results in a downward shift of anions’ orbital levels. As a result, the LUMO is dominated by the anions; thus, the anions are predominantly reduced and decomposed to form an anion-derived SEI. As for the formation mechanism of CEI, it has been widely reported that the components of CEI are mainly organic lithium salts in traditional diluted electrolytes. In this work, we found that the main component of CEI became LiF-based inorganic in concentrated electrolyte. The CEI formation mechanism in concentrated electrolyte is not yet clear, but we can draw on the research ideas of the SEI formation mechanism in concentrated electrolyte, from the perspective of HOMO levels, to study the effect of AGG on HOMO in concentrated electrolyte. This is also the main work we will do next. This is a very meaningful work, which helps to further understand the formation mechanism of CEI in concentrated electrolytes.

**10. In Figure 6a and 6b, if the colors are consistent (CH3O- and LiF2-), the readability would be better.**

Reply: We appreciate your suggestions. We carefully checked the original data and changed the colors in Figure 6b. The revised Fig 6 is shown below and has been revised in the manuscript

**Fig. 6** **TOF-SIMS spectra of interphases on the surface of cycled electrodes. a, b** normalized (to maximum) depth profiling of several typical second ion fragments on the cycled electrode outside surface; **c, d** TOF-SIMS chemical maps of several typical second ion fragments with 150s of sputtering on electrodes cycled in 1 M and 3 M electrolyte, respectively.

**11. It is claimed that “the higher intensity of FeF2-, FeF3-, MnF2-, and NiF3- fragments on the electrode surface cycled in 1 M electrolyte are presented clearly”. But in Figure 6 and Supplementary Figures 14-16, no any information of NiF3-.**

Reply:Thanks so much for this suggestion. We tested the relevant data together but did not present it. Now we present it in Supplementary Fig. 17. The revised Supplementary Fig. 17 is shown below and has been revised in the supplementary information.

**Supplementary Fig. 17.** Corresponding depth profiling of second ion fragments of interest on the electrode cycled in 1 M and 3 M electrolyte.

Reviewer#2:

Authors have implemented the changes as per the review comments to a satisfactory level. However, the manuscript needs to be thoroughly checked once by a native speaker for language corrections before publication.

Reply: Thank you very much for your valuable and constructive comments to improve the quality of this manuscript and our work. We have corrected the language carefully and thoroughly.

Reviewer #3

The authors have sufficiently addressed all my questions/comments in a good amount of details. The revised manuscript can be accepted for publication in Nat.Comm.

Reply: Thank you very much for your valuable and constructive comments to improve the quality of this manuscript and our work.

**Reviewer comments, third version:**

Reviewer #1 (Remarks to the Author):

All my questions/comments have been addressed. The revised manuscript can be accepted for publication in Nat.Comm.

**Author rebuttal, third version:**
